# NIH peer review: Criterion scores completely account for racial disparities in overall impact scores

**DOI:** 10.1126/sciadv.aaz4868

**Published:** 2020-06-03

**Authors:** Elena A. Erosheva, Sheridan Grant, Mei-Ching Chen, Mark D. Lindner, Richard K. Nakamura, Carole J. Lee

**Affiliations:** 1 Department of Statistics, Padelford Hall B-313, University of Washington, Seattle, WA 98195, USA.; 2 School of Social Work, 4101 15th Avenue NE, University of Washington, Seattle, WA 98195, USA.; 3 Center for Statistics and the Social Sciences, Padelford Hall C-14 University of Washington, Seattle, WA 98195, USA.; 4 Laboratoire J. A. Dieudonné, Université Côte d’Azur, CNRS, Nice, France.; 5 Center for Scientific Review, National Institutes of Health, 6701 Rockledge Drive, Bethesda, MD 20817, USA.; 6 Retired volunteer at Center for Scientific Review, National Institutes of Health, 6701 Rockledge Drive, Bethesda, MD 20817, USA.; 7 Department of Philosophy, Savery Hall 361, University of Washington, Seattle, WA 98195, USA.

## Abstract

Previous research has found that funding disparities are driven by applications’ final impact scores and that only a portion of the black/white funding gap can be explained by bibliometrics and topic choice. Using National Institutes of Health R01 applications for council years 2014–2016, we examine assigned reviewers’ preliminary overall impact and criterion scores to evaluate whether racial disparities in impact scores can be explained by application and applicant characteristics. We hypothesize that differences in commensuration—the process of combining criterion scores into overall impact scores—disadvantage black applicants. Using multilevel models and matching on key variables including career stage, gender, and area of science, we find little evidence for racial disparities emerging in the process of combining preliminary criterion scores into preliminary overall impact scores. Instead, preliminary criterion scores fully account for racial disparities—yet do not explain all of the variability—in preliminary overall impact scores.

## Teaser

Black-white disparities in preliminary peer review scores for NIH R01 grant applications are examined.

## INTRODUCTION

The National Institutes of Health (NIH) strives to fund the best grant applications—including applications from underrepresented minorities whose diverse perspectives enhance innovation and discovery in science and biomedical research (*1*–*5*). However, Ginther *et al*.’s groundbreaking studies (*6*–*8*) on NIH R01 applications for council years 2000–2006 demonstrated large funding disparities for black or African-American Principal Investigators (hereafter referred to as black PIs): The award probability for applications from black PIs was roughly 55% of that found for white PIs (16.1% versus 29.3%) (*8*), where a substantial portion of the variance in funding gap in applications from this period can be explained by differences in field-adjusted bibliometric measures (publications, citations, and journal impact factor) (*9*). Follow-up work by NIH on R01 applications from 2011 to 2015 focused on six decision points in the submission/resubmission and review process that could lead to differences in funding outcomes. They found that the funding gap remains, with racial disparities emerging in the selection of proposals for discussion by a study section, post-panel overall impact score assignment, and the tendency for black investigators to propose research on topics with lower award rates (*10*).

Psychological research demonstrates that increased ambiguity and uncertainty in evaluative contexts increases the expression of social bias (*11*–*15*). To diminish (though not eliminate) this, experts suggest scoring applications along a set of prespecified criteria to increase attention to factors related to merit (*16*, *17*). We might expect, then, that NIH’s introduction in 2009 of criterion scoring through its Enhanced Peer Review process—which was introduced to improve information and transparency for applicants (*18*)—would decrease the funding disparities between black and white PIs.

Under Enhanced Peer Review, for each application, the assigned reviewers (there are typically three) provide scores for the five criteria defined by the NIH—Significance, Investigator(s), Innovation, Approach, and Environment—and “derive” one preliminary overall impact score for each application. These preliminary criterion scores take integer values from 1 to 9, with 1 being the best, and (together with the preliminary overall impact score) are known as preliminary scores. NIH instructs reviewers to weigh the different criteria, as they see fit in deriving their overall impact scores (*19*), where an application “does not need to be strong in all categories to be judged likely to have major scientific impact” (*20*). Then, the averages of the preliminary overall impact scores determine which applications (roughly half) are selected for discussion at Scientific Review Group (SRG) meetings (*21*). After applications are discussed in the SRG meeting, all eligible reviewers record their final overall impact scores. When an assigned reviewer’s final overall impact score diverges from their preliminary overall impact score as a result of SRG discussion, they are asked to update their written critiques and criterion scores within 24 to 48 hours of the meeting for consistency. After SRG discussions, composite scores are calculated as the average of final overall impact scores from all eligible members of the SRG panel—not just the assigned reviewers—multiplied by 10; applicants sometimes refer to these composite scores as impact scores (*22*). Percentile scores calculated from the composite scores are then used as key inputs by NIH funding institutes for making funding decisions. Previous work has demonstrated that assigned reviewers’ final scores on all review criteria are related to final overall impact scores (*23*).

Recent work by Hoppe *et al*. found that the “decision point that makes the largest single contribution to the funding gap” is the selection of applications for discussion by a study section [(*10*), p. 6]. Our paper is the first to examine racial disparities in the assigned reviewer scores that precede and inform proposal selection for panel discussion. To begin, we evaluate whether racial disparities in NIH R01 funding remain under Enhanced Peer Review. Like Hoppe *et al*. (*10*), we find substantial funding gaps between black and white applicants. We then examine the relationship between assigned reviewers’ preliminary criterion scores and preliminary overall impact scores to evaluate the hypothesis that there are black-white differences in how preliminary criterion scores are combined to produce preliminary overall impact scores. This hypothesis about the presence of commensuration bias (*24*, *25*) is motivated by psychological research demonstrating that, in the calculation of overall scores, criteria scores can be aggregated in ways that favor members of preferred social groups (*11*–*15*). Furthermore, we investigate whether race-related disparities in preliminary overall impact scores can be explained by differences in preliminary criterion scores and/or by their commensuration (*26*) into preliminary overall impact scores. To study these questions, we use multilevel modeling on assigned reviewers’ preliminary scores, which, unlike final scores, are assigned to all applications. We find some evidence of black-white differences in commensuration practices with respect to individual criteria. However, the combined effect of these commensuration differences on the preliminary overall impact scores is practically and statistically negligible. At the same time, we demonstrate that preliminary criterion scores fully account for racial disparities—yet come short of explaining all of the variability—in preliminary overall impact scores. Overall, we conclude that preliminary criterion scores absorb rather than mitigate racial disparities in preliminary overall impact scores.

### NIH reviews are inherently multilevel

**Fig. 1:**
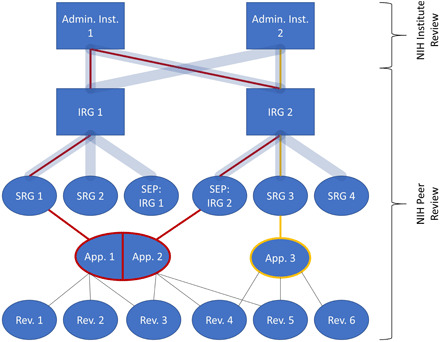
Multilevel NIH review structure for a hypothetical example of three applications (App. 1, 2, and 3) submitted by two PIs (yellow and red). Thick blue lines show structural connections. Thin lines show hypothetical assignments for the three applications. Rectangles are specified as fixed effects and ellipses as random effects in our mixed-effects models.

Assigned reviewers’ preliminary scores represent the very first step in the NIH’s grant review process. The scientific merit of applications is evaluated within SRGs (study sections) that are organized within Integrated Review Groups (IRGs) by general scientific area (*21*). In addition, within IRGs, Special Emphasis Panels are formed to review other topics and member conflict applications (*27*). NIH funding (administering) institutes carry out a second round of review and ultimately make funding decisions (*28*). Individual PIs may submit applications to different SRGs; reviewers review multiple applications within an SRG and may review for more than one IRG/SRG. Figure 1 provides an example diagram of the NIH review structure.

## MATERIALS AND METHODS

We use the IMPAC II (Information for Management, Planning, Analysis, and Coordination) grant data system, which stores information about each NIH application and self-reported demographics such as race and gender. Study variables include preliminary overall impact and preliminary criterion scores, structural covariates (indicators for IRG, SRG, administering institute, application, applicant, and reviewer), and other applicant- and application-specific covariates, summarized in Table 1. Applicant- and application-specific characteristics were chosen to include variables that were previously shown to affect overall impact scores net of criterion scores—council year, age group, and human and animal subject codes (*29*). NIH’s descriptions for the criterion and overall impact scores can be found in Table 2.

**Table 1:** Study variables. IPEDS, Integrated Postsecondary Education Data System, a database of survey information gathered by the Department of Education about every college or university that participates in federal financial aid programs; HBCU, historically black college or university; HSI, Hispanic-serving institution; SEP, Special Emphasis Panel. See model descriptions for variable inclusion.

**Type**	**Name**	**Description**
Dependent variable	Preliminary overall impact	Integer score from 1 to 9; smaller is better
Variables of interest		
Race	PI black	1 for black, 0 for white; self-reported
Preliminary criteria		
	Significance	Integer score from 1 to 9; smaller is better
	Investigator	Integer score from 1 to 9; smaller is better
	Innovation	Integer score from 1 to 9; smaller is better
	Approach	Integer score from 1 to 9; smaller is better
	Environment	Integer score from 1 to 9; smaller is better
Structural covariates		
CSR peer review		
	IRG	Integrated Review Group
	SRG	Scientific Review Group
	Institute/Center	NIH Institute/Center making funding decisions
Other indicators		
	Application ID	Encrypted application indicator
	Applicant ID	Encrypted applicant/PI indicator
	Reviewer ID	Encrypted reviewer indicator
Other covariates		
Applicant-specific		
	Gender	F/M, self-reported
	Ethnicity	Hispanic/Latino or not, self-reported
	Career stage	Early stage (ES), experienced, or non-ES new investigator
	Degree type	Ph.D., M.D., M.D./Ph.D., Other
	Terminal degree year	Year of most recent degree
	NIH funding history	First NIH application, previously applied, or previously funded
	Geographic location	Location of institution: central, east, south, or west
	NIH funding bin	FY 2014 total institution NIH funding; five bins
	Institution sector	Public, private, or other
	Graduate education	1 if institution provides graduate education; 0 if not
	IPEDS lookup	1 if institution in IPEDS database; 0 if not
	MSI type	Minority-serving institution type: HBCU, HSI, or otherwise
Application-specific		
	Application type	New or renewal
	Solicitation type	Request for application, Program announcement, Others
	Amended status	Amended or not
	Multiple PIs	Yes or no
	Requested costs	Funding dollars requested
	Support years	Support years requested, from 1 to 5
	Council year	2014–2016; year of review councils
	Review group type	Standing study section, recurring SEP, or nonrecurring SEP
	Human subjects	Acceptable, unacceptable, or inapplicable
	Animal subjects	Acceptable, unacceptable, or inapplicable
	Child code	Acceptable, unacceptable, or inapplicable
	Gender code	Acceptable, unacceptable, or inapplicable
	Minority code	Acceptable, unacceptable, or inapplicable

**Table 2:** NIH’s descriptions for overall impact and five review criteria (*48*).

**Score**	**Description**
Overall impact	Reviewers will provide an overall impact/priority score to reflect their assessment of the likelihood for the project to exert a sustained, powerful influence on the research field(s) involved, in consideration of the following five core review criteria, and additional review criteria (as applicable for the project proposed).
Scored review criteria	
Significance	Does the project address an important problem or a critical barrier to progress in the field? If the aims of the project are achieved, how will scientific knowledge, technical capability, and/or clinical practice be improved? How will successful completion of the aims change the concepts, methods, technologies, treatments, services, or preventative interventions that drive this field?
Investigators	Are the PIs, collaborators, and other researchers well suited to the project? If early-stage investigators or new investigators are in the early stages of independent careers, do they have appropriate experience and training? If established, have they demonstrated an ongoing record of accomplishments that have advanced their field(s)?
Innovation	Does the application challenge and seek to shift current research or clinical practice paradigms by using novel theoretical concepts, approaches or methodologies, instrumentation, or interventions? Are the concepts, approaches or methodologies, instrumentation, or interventions novel to one field of research or novel in a broad sense? Is a refinement, improvement, or new application of theoretical concepts, approaches or methodologies, instrumentation, or interventions proposed?
Approach	Are the overall strategy, methodology, and analyses well reasoned and appropriate to accomplish the specific aims of the project? Are potential problems, alternative strategies, and benchmarks for success presented? If the project is in the early stages of development, will the strategy establish feasibility and will particularly risky aspects be managed?
Environment	Will the scientific environment in which the work will be done contribute to the probability of success? Are the institutional support, equipment, and other physical resources available to the investigators adequate for the project proposed? Will the project benefit from unique features of the scientific environment, subject populations, or collaborative arrangements?

This study considered a full set of 54,740 R01 applications submitted by black and white PIs and reviewed by NIH’s Center for Scientific Review (CSR) during council years 2014–2016. CSR reviews about 90% of the R01 applications; applications submitted to funding opportunity announcements with special review criteria are sometimes managed by the funding institutes. A total of 1771 applications submitted by PIs whose race was American Indian or Alaskan, Asian, Native Hawaiian, or Pacific Islander or by PIs who indicated more than one race, as well as 8648 applications for which PI’s race was withheld or unknown, were excluded from the study. At the time of application, PI demographics are voluntarily reported by applicants; NIH requests but cannot compel PIs to provide this information. Self-reported demographics do not appear with the application when it is handled by reviewers or by the NIH review committee, staff, or council, although race might be known from personal knowledge or inferred from information available on the internet or in the application materials (e.g., name, receipt of a minority fellowship/grant, or other NIH biosketch content). Approximately 15% of the applications from black and white PIs were missing information on PI gender, ethnicity (Hispanic/Latino or not), and degree and were excluded from the study. The remaining 46,226 applications—1015 (or 2.2%) from black PIs and 45,211 (or 97.8%) from white PIs—were evaluated by 19,197 unique reviewers who wrote 139,216 reviews (table S1). More details about the data are available in the “Study data” section in the Supplementary Materials.

Because of the sensitive nature of NIH peer review records, study data were sampled from a full set of 54,740 R01 applications submitted by black and white PIs and reviewed by NIH’s CSR during council years 2014–2016. Given the relatively low representation of black investigators among NIH applicants, our primary analyses rely on a matched subset where applications from black applicants are matched to applications from white applicants (hereafter referred to as “matched black” and “matched white” applications).

We used exact matching on eight key variables thought to be related to scores and award rates. Exact matching can be considered a version of coarsened exact matching (*30*) with complete matching on selected variables and full coarsening on other variables (a proof is available in the “Coarsened exact matching with exact matching on a subset of covariates” section in the Supplementary Materials). The matching variables, summarized in Table 3, are contact PI’s gender, ethnicity, career stage, degree type, institution’s NIH funding bin, application type, application’s amended status (first submission or resubmission), and the area of science as represented by the IRG. The funding bins—with 20% of black applications in each bin—were defined by ordering the 1015 black applications by total NIH funding received by the applicant’s institution in fiscal year (FY) 2014 (see table S2). The selection of matched white applications was done subject to the constraint that no individual reviewer can have more than four reviews in the sample to ensure the privacy and confidentiality of reviewers. Matches were found for 890 of the 1015 black applications, which is more than 87% (Table 4). Our matching procedure improved balance on all the matching variables and on most other applicant- and application-specific covariates (table S3). The improved balance makes estimates from the matched subset analysis more robust, or less susceptible to model misspecification, than analyses based on a random sample (*31*, *32*). The “Study data” section in the Supplementary Materials provides further details on the matching and on evaluating the efficacy of the matching in improving balance.

**Table 3:** Matching variables.

**Name**	**Description**
Applicant	
Gender	F/M, self-reported
Ethnicity	Hispanic/Latino or not, self- reported
Career stage	Early stage (ES), experienced, or non-ES new investigator
Degree type	Ph.D., M.D., M.D./Ph.D., other
NIH funding bin	FY 2014 total institution NIH funding; five bins
Application	
Application type	New or renewal
Amended status	Amended or not
IRG	Integrated Review Group

**Table 4:** Sampled data summary statistics by application subset.

**Subset**	**Unique PIs**	**Reviewers**	**Reviews**	**Applications**
All black	500	2,310	2,926	1,015
Matched black	456	2,084	2,578	890
Matched white	1,497	3,866	4,893	1,676
Random white	1,904	4,460	5,669	2,030
Total	3,679	7,901	13,140	4,596

In addition to our main analysis of matched data, for comparative purposes, we repeated our analyses for a random sample in which applications from black applicants were compared with randomly selected applications from white applicants, hereafter referred to as “random white” applications. The “Random subset selection” section in the Supplementary Materials provides details about how the random white applications were chosen. Our main results for the matched subset, presented here, were largely confirmed by our analyses of the random subset (see the “Random subset analyses” section in the Supplementary Materials).

Last, because of the sensitive nature of individual-level data, only a limited dataset that maintains privacy and confidentiality in compliance with NIH policy is available for public use. We provide the URL of the public-use data depository in the Acknowledgments section. This public-use dataset includes the same reviews and most of the study’s main variables but fewer covariates. For reproducibility purposes, we repeated the main analyses on the public-use dataset (see the “Reproducibility” section in the Supplementary Materials).

### Multilevel modeling

For multilevel modeling of review scores, we relied on the NIH review structure (Fig. 1), distinguishing between structural variables and other covariates that could potentially be associated with preliminary overall impact scores. IRG, SRG, and administering institute, as well as reviewer and PI indicators, are structural variables, as they represent various levels of clustering in the data. All of our models account for structural dependencies in the data via the inclusion of fixed effects for IRG and administering institute and random effects for SRG, reviewer, and PI indicators; the fixed effects are marked with rectangles and random effects with ellipses in Fig. 1. Application ID was not included in any models, because the PI ID random effect captured nearly all variability in application ID. Note that individual differences between reviewers—reflected by the reviewer random intercept in our models—can be thought of as being due to individual differences in areas of expertise, scientific interests, and value systems (*33*, *34*). Likewise, individual differences between PIs are reflected by the PI random intercept in our models, and average differences in preliminary overall impact scores between SRGs are captured by the SRG random effects. Other covariates include the applicant- and application-specific covariates from Table 1. Last, the five preliminary criterion scores can also be thought of as additional covariates that explain variability in preliminary overall impact scores. See the Supplementary Materials for further discussion of the hierarchical structure specification.

Let *Y_ijklm_* be the preliminary overall impact score for the *i*th review of the *j*th application from the *k*th PI (reviewed by the *l*th reviewer in the *m*th SRG), *R_k_* a race indicator (1 indicates a black PI), and *X_jk_* a vector of application- and applicant-specific control variables. To estimate racial disparities, we consider the following mixed effects model formulation

$$Y_{\text{ijklm}}=\alpha +\beta_{R}R_{k}+\beta X_{\text{jk}}+\gamma_{k}+\xi_{l}+\eta_{m}+\varepsilon_{\text{ij}}$$

where α is the model intercept; β_R_ is the race coefficient; β is the vector of coefficients for control variables; γ*_k_*, ξ*_l_*, and η*_m_* are random intercepts for PI, reviewer, and SRG; and ε*_ij_* are within-application independent Gaussian error terms. For more information about the rationale and tests for the random effects specification, see the “Model specifications” section in the Supplementary Materials. We examine estimates of the race coefficient β_R_ from a series of models, first only adjusting for the structural covariates and then including applicant- and application-level characteristics and preliminary criterion scores among the control variables *X* (see Table 5).

**Table 5:** Selected parameter estimates from models 1 to 4. Race coefficient estimates, their effect sizes, and variance components estimates from four hierarchical linear models for preliminary overall impact scores fit on *n* = 7471 reviews of 2566 applications. Model 1 controls for structural covariates; model 2 controls for structural and applicant/application-specific covariates; model 3 controls for structural covariates and criterion scores; model 4 controls for structural and applicant/application-specific covariates and criterion scores. Control variables are listed in Table 1. Coefficient estimates for control variables are not shown. Significance * is reported for *P* < 0.005. In mixed-effects models, multiple effect sizes exist for a given coefficient; we report the coefficient divided by the residual SD. For more information, see (*49*).

**Parameters**	**Model 1**	**Model 2**	**Model 3**	**Model 4**
Race fixed effect				
Coefficient	0.466*	0.350*	0.010	0.014
(SE)	(0.062)	(0.051)	(0.017)	(0.018)
*P*	<0.005	<0.005	0.561	0.431
Effect size	0.358	0.272	0.018	0.025
Random effects				
Reviewer SD	0.507	0.500	0.286	0.286
PI SD	0.883	0.578	0.100	0.082
SRG SD	0.343	0.271	0.084	0.075
Residual SD	1.300	1.284	0.565	0.562

To study commensuration practices, we focus on interaction effects between race and preliminary criterion scores. Let *Z_ij_* be the vector of preliminary criterion scores associated with the *i*th review of the *j*th application. The linear commensuration model for the preliminary overall impact score *Y_ijklm_* of the *i*th review of the *j*th application from the *k*th PI (reviewed by the *l*th reviewer in the *m*th SRG) is specified by

$$Y_{\text{ijklm}}=\alpha +\beta_{R}R_{k}+\beta_{C}Z_{\text{ij}}+\beta_{I}R_{k}Z_{\text{ij}}+\beta X_{\text{jk}}+\gamma_{k}+\xi_{l}+\eta_{m}+\varepsilon_{\text{ij}}$$

where α is the model intercept; β_R_ is the race coefficient; β_C_ is a vector of preliminary criterion score coefficients; β*_I_* is the vector of commensuration coefficients for the interactions between race and preliminary criterion scores; β is the vector of coefficients for control variables *X_jk_*; γ*_k_*, ξ*_l_*, and η*_m_* are random intercepts for PI, reviewer, and SRG; and ε*_ij_* are within-application independent Gaussian error terms. For commensuration models, the control variables *X* include structural and applicant- and application-level characteristics. See the “Commensuration practices” section in the Supplementary Materials for details on interpretation.The University of Washington team performed the analyses. The University of Washington’s Institutional Review Board determined that the study did not involve human subjects.

## RESULTS

### Award rates

First, we compare award rates for black, matched white, and random white applicants to see whether there is a funding gap between black and white applicants and, if so, to determine whether matching on key characteristics including the area of science eliminates the funding gap. Overall, for CSR-reviewed R01 applications from black and white investigators for council years 2014–2016, the award probability for black applications was 55% of that for white applications (10.2% versus 18.5%). Our sampled dataset, summarized in Table 4, includes applications from investigators with Ph.D.’s, M.D.’s, and M.D.’s/Ph.D.’s. In these sampled data, the award rate for black applications was approximately 56% of that of random white applications (11.03% versus 19.66%). After matching on the variables listed in Table 3, we find the award rate for matched black applications to be 75% of that for matched white applications (11.57% versus 15.39%). Thus, matching on variables that include area of science as represented by the IRG reduces the award disparity between black and white applications by 56%. Because funding disparities for black applications are driven by disparities in peer review scores (*6*–*8*, *10*), we now examine the assigned reviewers’ preliminary overall impact scores.

### Racial disparity in preliminary overall impact scores

**Fig. 2:**
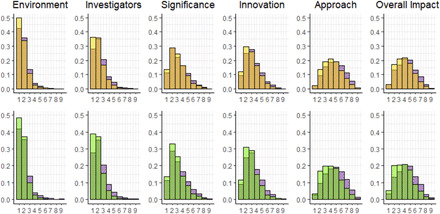
Frequency histograms for the five preliminary criterion scores and the preliminary overall impact score. Top row: matched black (purple) and matched white (yellow) applications comparison, with overlap in orange; bottom row: all black (purple) and random white (light green) applications comparison, with overlap in dark green.

Comparisons between the histograms of preliminary overall impact scores (ranging from 1 to 9) for black and white applications demonstrate that matched white applications tend to receive better (lower) scores than black applications (Fig. 2, top right) and that this difference is more pronounced for the comparison with random white applications (Fig. 2, bottom right). Controlling for structural variables—IRG, SRG, and administering institute, as well as reviewer and PI indicators—we estimate that the average difference in preliminary overall impact scores between black and random white applications is 0.700 points (table S5).

Next, we use linear mixed-effects regression models (*35*, *36*) to evaluate whether racial disparities in preliminary overall impact scores of assigned reviewers can be explained by other application and applicant characteristics and the hypothesized commensuration practices. To estimate racial disparities in preliminary overall impact scores, we distinguish between controlling for structural variables that are related to NIH’s review structure in Fig. 1, other covariates (applicant- and application-level) that can potentially be associated with preliminary overall impact scores, and preliminary criterion scores. Multilevel modeling accounts for the internal structure of NIH grant reviews, yielding two key analytical advantages. First, it provides correct estimates of the SEs of model coefficients by appropriately accounting for the complex network of dependencies between reviews. Second, it allows us to compare sources of variability in preliminary overall impact scores directly. See the “Multilevel modeling” section in the Supplementary Materials for more details.

Here, we present results from multilevel analyses for the matched subset, which is less susceptible to model misspecification (*31*, *32*). Results for the random subset analysis are provided for comparison in the Supplementary Materials (table S5). Last, the “Reproducibility” section in the Supplementary Materials provides analogous results obtained on the public-use dataset (table S9).

Table 5 provides estimates of racial disparities in preliminary overall impact scores, controlling for structural and other covariates. To indicate statistical significance, we use the recommended 0.005 *P* value cutoff for “new discoveries” (*37*). For practical significance, we argue that a difference of 0.3 points or more in overall impact score for applications near the funding cutoff is substantial. For example, at the 15th percentile of our sampled data, increasing (or decreasing) an application’s final overall impact score by 0.3 points moves that application, on average, up to the 20th (or down to the 12th) percentile. Because NIH award rates are low—typically between 10 and 20%—differences as little as 0.3 points in the overall impact score could tangibly affect funding decisions.

For the matched subset analysis (Table 5), we find that there is a statistically significant difference of 0.466 points in the average preliminary overall impact scores between black and white applicants when we only account for structural dependencies, including the area of science (model 1). This difference decreases to 0.350 points, but remains statistically significant, when we also control for applicant- and application-level characteristics (model 2). However, the difference becomes practically and statistically negligible when preliminary criterion scores are included as control variables in addition to the applicant- and application-level characteristics (model 4).

From Table 5, examining the unexplained variability in preliminary overall impact scores, we see that, while the estimate of residual SD in model 2 (1.284) is essentially the same as that in model 1 (1.300), it decreases markedly to 0.562 points (model 4) after preliminary criterion scores are included. This indicates that preliminary criterion scores play a major role in describing variability in preliminary overall impact scores, although they are not able to explain it fully. Notice also that adding preliminary criterion scores (model 4) markedly reduces the estimated variability in preliminary overall impact scores that is due to PI (SD for PI random effects is reduced nearly 10-fold).

Importantly, when we include only preliminary criterion scores in addition to structural covariates (model 3; Table 5), we find no significant racial disparity in preliminary overall impact scores, as is the case for model 4, which adjusts for various applicant- and application-level characteristics in addition to preliminary criterion scores. Note also that estimates of variance components from model 3 are nearly identical to those from model 4. We find that, after controlling for preliminary criterion scores, the disparity in preliminary overall impact scores between black and white applications becomes just 0.01 points—which is negligible, practically and statistically—whether or not one controls for other application- and applicant-specific covariates. Repeating these analyses for the random subset (see table S5), we also find that preliminary criterion scores alone explain essentially all of the racial disparity in preliminary overall impact scores.

Focusing on preliminary criterion scores, we see systematic racial differences (Fig. 2). The disparity is largest for Approach score, with a mean of 4.75 for black applications and 4.12 for random white applications (*P* < 0.005). Approach is the criterion weighed most heavily in determining the preliminary overall impact score in our analyses, as well as in previous research on final scores (*29*).

### Commensuration model for preliminary overall impact scores

To examine our motivating question about differences in how reviewers weigh preliminary criterion scores when deciding preliminary overall impact scores, we control for all structural and application- and applicant-specific characteristics and estimate the key first-order commensuration coefficients—the interactions between the race indicator and the preliminary criterion scores—for the matched subset of the data. Table 6 contains relevant parameter estimates from the linear commensuration model; estimates for other control variables are not shown. Results for the random subset analysis are provided in the Supplementary Materials for comparison (table S6). The “Reproducibility” section in the Supplementary Materials provides analogous commensuration model results obtained on the public-use dataset (table S10).

**Table 6:** Selected parameter estimates, commensuration model. Preliminary criterion score, race, and commensuration coefficient estimates, and variance components estimates, for preliminary overall impact scores on *n* = 7471 reviews of 2566 applications. Control variables (coefficient estimates not shown) include structural and applicant/application-specific covariates from Table 1. Significance * is reported for *P* < 0.005.

**Variable**	**Estimate (SE)**	***P***
Fixed effects		
Significance	0.258* (0.008)	<0.005
Investigator	0.057* (0.011)	<0.005
Innovation	0.129* (0.008)	<0.005
Approach	0.598* (0.007)	<0.005
Environment	0.022 (0.011)	0.057
PI race = black	−0.024 (0.047)	0.610
Significance * PI black	−0.034 (0.013)	0.010
Investigator * PI black	0.018 (0.017)	0.298
Innovation * PI black	−0.020 (0.014)	0.144
Approach * PI black	0.041* (0.012)	<0.005
Environment * PI black	−0.010 (0.018)	0.596
Random effects		
Reviewer intercepts SE	0.286	
PI intercepts SE	0.079	
SRG intercepts SE	0.076	
Residual variability SE	0.562	

Interpretation of race and criteria effects becomes more complicated when their interactions are included in the model. Significant interaction terms in Table 6 indicate commensuration differences: The effect of preliminary criterion scores on the preliminary overall impact score depends on applicant race. Using the *P* = 0.005 cutoff for new discoveries (*37*), we find that the contribution of the preliminary Approach score to the preliminary overall impact score is higher (worse) for black applications (the interaction coefficient is 0.041; *P* < 0.005) as compared to matched white applications. The statistically significant relationship between the preliminary Approach score and the preliminary overall impact score as estimated by the model is such that black applications appear to be “penalized” for Approach. Notice that negative estimates for interaction coefficients in Table 6 are suggestive of black applicants being “rewarded” for those aspects; however, these estimates do not reach 0.005 statistical significance. Overall, for the preliminary overall impact score, we find that the combined extent and magnitude of commensuration differences across all criterion scores are not large. Estimated expected differences in the overall score of 0.1 points or more as a result of commensuration differences are rare, and the change of 0.1 is small relative to the variability due to other sources (see the “Commensuration practices” section in the Supplementary Materials and fig. S1). This finding was confirmed on the random subset analysis (fig. S2) and reproduced with the public-use dataset (figs. S3 and S4).

### Final (post-discussion) overall impact scores

Of the assigned reviewers who change their overall impact scores after discussion, only 43% recorded respective changes in their criterion scores (see table S7); it is unknown why some reviewers change their criterion scores and others do not. Examining reviewer scores provided by the assigned reviewers after discussion, we find variability in reviewer random effects and residual variability to be considerably lower for post-discussion than for preliminary scores. This is consistent with the idea that panel discussions lead reviewers toward consensus (*38*). Our conclusions regarding racial disparity for final overall impact scores are largely the same as for preliminary overall impact scores: Final criterion scores fully explain racial disparity in final overall impact scores between white and black applicants in the matched subset (see table S8). We further note that final (post-discussion) scores are unsuitable for analyzing differences in commensuration because commensuration asymmetries are conceptualized as happening at the individual reviewer level (*24*, *25*) and—unlike preliminary scores that represent individual reviewer evaluations—final scores also reflect SRG discussions.

## DISCUSSION

We find that, in the R01 applications for black and white investigators from 2014 to 2016, the overall award rate for black applications is 55% of that for white applications (10.2% versus 18.5%), resulting in a funding gap of 45%. This funding gap is substantial, although it—like the gap found by Hoppe *et al*. (*10*)—cannot be directly compared to the previously reported gap in NIH grant review (*6*–*8*) before NIH introduced scored criteria to increase information and transparency to its applicants (*18*). Direct comparisons are not possible due to procedural differences in the peer review process (before and after Enhanced Peer Review) as well as methodological differences, which include, for example, the use of self-reported race alone in this study as opposed to self-reported race and information supplemented from the Association of American Medical Colleges Faculty Roster in the Ginther *et al*. studies (*6*–*8*). The funding gap remains despite psychological research, suggesting that using scored individual criteria can focus attention on merit-related factors and decrease bias in expert judgment under complex evaluative conditions (*16*, *17*, *39*). We find that the black/white funding gap decreases to 25% after matching. Matched applications with exact matches on gender, ethnicity (Hispanic/Latino or not), career stage, type of academic degree, institution prestige (as reflected by the NIH funding bin), area of science (as reflected by the IRG handling the application), and application type (new or renewal) and status (amended or not) have award rates of 11.57% for matched black versus 15.39% for matched white. Likewise, examining application scores, we find that our matching procedure reduces the gap in preliminary overall impact scores between black and while applications by one-third, from a 0.700- to 0.466-point difference.

Note that, unlike previous work on race and NIH R01 funding (*6*–*8*, *10*), our main analyses rely on individual reviewer–level preliminary scores from all applications, discussed or not. All estimates reported in this paper from multilevel models control for variables reflecting the structure of NIH reviews including general area of science (via NIH IRG, SRG, and Institute/Center) and reviewer and PI indicators.

Without controlling for criterion scores, we estimate that matched black applications have preliminary overall impact scores that are, on average, 0.466 points worse than those of matched white applications (model 1; Table 5). Controlling for applicant-specific (e.g., gender, ethnicity, degree type, terminal degree year, and NIH funding history) and application-specific (e.g., requested direct costs, resubmission versus original submission, and subject codes) covariates reduces this gap to 0.350 points (model 2; Table 5), a difference that can still be important for applications that are competitive for funding (see the “Multilevel modeling” section in the Supplementary Materials).

Controlling for criterion scores, on the other hand, completely accounts for the difference associated with race in preliminary (and final) overall impact scores. Therefore, we conclude that preliminary criterion scores absorb rather than mitigate racial disparities in preliminary overall impact scores in NIH grant review. This conclusion is notable, because overall scores are far from being completely determined by criterion scores: They come short in explaining reviewer and residual variability especially. This conclusion is based on observed associations and does not support or imply causal relationships: In particular, it does not assume that after exact matching on eight key variables thought to be related to scores and award rates (Table 3), reviewers follow a procedure whereby they first assign criterion scores and then derive an overall impact score. At the same time, we find little evidence for racial disparities emerging in the process of combining preliminary criterion scores into preliminary overall impact scores.

Limitations of our study point to future research directions. First, missing data on demographic characteristics deserve further attention. Our study only had access to applications with complete demographic information; in addition, 15% of the applications from black and white PIs were missing information on PI gender, ethnicity (Hispanic/Latino or not), or degree and were excluded from the study (see the “Study data” section in the Supplementary Materials for more details). Second, our study focused on examining the relationship between preliminary criterion and preliminary overall impact scores and did not scrutinize other steps in NIH review such as the advancement of applications from preliminary review to SRG discussion. Last, while our study contains a number of important applicant- and application-level variables such as the applicant’s time since degree, the amount of NIH funding received by the applicant’s institution, and the applicant’s NIH funding history (see Table 1 for the full list), there are others that could be influential. In particular, we do not have finer-grained information about PI topic choice. Recent work suggests that topic choice could create a vicious cycle where investigators’ preference for topics “less likely to excite the enthusiasm of the scientific community” could lead to lower funding rates, “which in turn limits resources and decreases the odds of securing funding in the future” [(*10*), p. 8]. Nor do we have bibliometric profiles or mentorship network measures for the applicants. Although numbers of publications and citations may not be appropriate measures of productivity either for investigators or for grant awards—a number of studies suggest that rigorous and innovative research projects will produce a wide range of bibliometric outputs and an overemphasis on bibliometrics may actually discourage rigor and innovation (*40*–*43*)—bibliometrics have been found to explain a substantial portion of the black/white R01 funding gap (*9*). Likewise, underrepresented researchers were found to have smaller intra-institutional coauthor networks, which were associated with lower publication and citation counts (*44*). While omitted variable bias could, in theory, pose a problem, in our case it seems unlikely because—with preliminary criterion scores in the model—the estimated race coefficient remains virtually unchanged whether available applicant- and application-specific variables are included in the model or not.

More research is necessary to understand the reasons behind differences in preliminary criterion scores between black and white NIH R01 applications. We find that black investigators, on average, receive worse preliminary scores on all five criteria—Significance, Investigator(s), Innovation, Approach, and Environment—even after matching on key variables that include career stage, gender, degree type, and area of science (Fig. 2). This finding is consistent with multiple explanations that are not incompatible: implicit racial preferences (*45*), which may get expressed more strongly when evaluators have more discretion to interpret, apply, and prioritize criteria (*11*–*13*, *15*, *46*); black PIs disproportionately pursuing research in areas on which reviewers may not place a high priority (*10*); black-white differences in research productivity or impact (*9*); and/or the cumulative effect of disparities experienced over a PI’s academic career including differences in mentorship and social networks (*8*, *9*, *44*, *47*). Future research should evaluate the extent to which these possibilities account for racial disparities in preliminary criterion scores.

## Supplementary Material

aaz4868_SM.pdf

## SUPPLEMENTARY MATERIALS

Supplementary material for this article is available at http://advances.sciencemag.org/cgi/content/full/6/23/eaaz4868/DC1

## References

[1] National Research Council, *Research Universities and the Future of America: Ten Breakthrough Actions Vital to Our Nation’s Prosperity and Security* (National Research Council, 2012).

[2] National Academy of Engineering, *Engineering Research and America’s Future: Meeting the Challenges of a Global Economy* (National Academy of Engineering, 2005).

[3] National Academy of Sciences, National Academy of Engineering, and and Institute of Medicine, *Expanding Underrepresented Minority Participation: America’s Science and Technology Talent at the Crossroads* (National Academies Press, 2010).

[4] Tabak L. A., Collins F. S., Weaving a richer tapestry in biomedical science. Science 333, 940–941 (2011). 21852476 10.1126/science.1211704 PMC3440455

[5] Valantine H. A., Collins F. S., National Institutes of Health addresses the science of diversity. Proc. Natl. Acad. Sci. U.S.A. 112, 12240–12242 (2015). 26392553 10.1073/pnas.1515612112 PMC4603507

[6] Ginther D. K., Haak L. L., Schaffer W. T., Kington R., Are race, ethnicity, and medical school affiliation associated with NIH R01 type 1 award probability for physician investigators? Acad. Med. 87, 1516–1524 (2012). 23018334 10.1097/ACM.0b013e31826d726b PMC3485449

[7] Ginther D. K., Kahn S., Schaffer W. T., Gender, race/ethnicity, and National Institutes of Health R01 research awards: Is there evidence of a double bind for women of color? Acad. Med. 91, 1098–1107 (2016). 27306969 10.1097/ACM.0000000000001278 PMC4965301

[8] Ginther D. K., Schaffer W. T., Schnell J., Masimore B., Liu F., Haak L. L., Kington R., Race, ethnicity, and NIH research awards. Science 333, 1015–1019 (2011). 21852498 10.1126/science.1196783 PMC3412416

[9] Ginther D. K., Basner J., Jensen U., Schnell J., Kington R., Schaffer W. T., Publications as predictors of racial and ethnic differences in NIH research awards. PLOS ONE 13, e0205929 (2018). 30427864 10.1371/journal.pone.0205929 PMC6235266

[10] Hoppe T. A., Litovitz A., Willis K. A., Meseroll R. A., Perkins M. J., Hutchins B. I., Davis A. F., Lauer M. S., Valantine H. A., Anderson J. M., Santangelo G. M., Topic choice contributes to the lower rate of NIH awards to African-American/black scientists. Sci. Adv. 5, eaaw7238 (2019). 31633016 10.1126/sciadv.aaw7238 PMC6785250

[11] Hodson G., Dovidio J. F., Gaertner S. L., Processes in racial discrimination: Differential weighting of conflicting information. Pers. Soc. Psychol. Bull. 28, 460–471 (2002).

[12] Norton M. I., Sommers S. R., Vandello J. A., Darley J. M., Mixed motives and racial bias: The impact of legitimate and illegitimate criteria on decision making. Psychol. Public Policy Law 12, 36–55 (2006).

[13] Norton M. I., Vandello J. A., Darley J. M., Casuistry and social category bias. J. Pers. Soc. Psychol. 87, 817–831 (2004). 15598108 10.1037/0022-3514.87.6.817

[14] Uhlmann E., Cohen G. L., Constructed criteria: Redefining merit to justify discrimination. Psychol. Sci. 16, 474–480 (2005). 15943674 10.1111/j.0956-7976.2005.01559.x

[15] Uhlmann E. L., Cohen G. L., “I think it, therefore it’s true”: Effects of self-perceived objectivity on hiring discrimination. Organ. Behav. Hum. Decis. Process. 104, 207–223 (2007).

[16] W. Thorngate, R. M. Dawes, M. Foddy, *Judging Merit* (Psychology Press, 2009).

[17] D. Kahneman, *Thinking, Fast and Slow Farrar* (Straus and Giroux, ed. 1, 2013).

[18] NIH Staff, *Get a Handle on Changes from the Enhancing Peer Review Process* (NIH, 2009).

[19] NIH Staff, *Scoring Guidance* (NIH, 2016).

[20] NIH Staff, *Scoring System and Procedure* (NIH, 2012).

[21] NIH Staff, *Integrated Review Groups | NIH Center for Scientific Review* (NIH, 2019).

[22] NIH Staff, *Peer Review* (NIH, 2018).

[23] Lindner M. D., Vancea A., Chen M. C., Chacko G., NIH peer review: Scored review criteria and overall impact. Am. J. Eval. 37, 238–249 (2016). 27239158 10.1177/1098214015582049 PMC4882120

[24] Lee C. J., Commensuration bias in peer review. Philos. Sci. 82, 1272–1283 (2015).

[25] White House Staff, *Implementation of Federal Prize Authority: Fiscal Year 2014 Progress Report* (Obama White House, 2015).

[26] Espeland W. N., Stevens M. L., Commensuration as a social process. Annu. Rev. Sociol. 24, 313–343 (1998).

[27] NIH Staff, *All Other CSR Special Emphasis Panels | NIH Center for Scientific Review* (NIH, 2019).

[28] NIH Staff, *List of NIH Institutes, Centers, and Offices* (NIH, 2015).

[29] Eblen M. K., Wagner R. M., RoyChowdhury D., Patel K. C., Pearson K., How criterion scores predict the overall impact score and funding outcomes for National Institutes of Health peer-reviewed applications. PLOS ONE 11, e0155060 (2016). 27249058 10.1371/journal.pone.0155060 PMC4889138

[30] Iacus S. M., King G., Porro G., Causal Inference without balance checking: Coarsened exact matching. Polit. Anal. 20, 1–24 (2012).

[31] Erosheva E., Walton E. C., Takeuchi D. T., Self-rated health among foreign- and US-born asian americans: A test of comparability. Med. Care 45, 80–87 (2007). 17279024 10.1097/01.mlr.0000241114.90614.9c PMC2773448

[32] Ho D. E., Imai K., King G., Stuart E. A., Matching as nonparametric preprocessing for reducing model dependence in parametric causal inference. Polit. Anal. 15, 199–236 (2007).

[33] Hargens L. L., Herting J. R., Neglected considerations in the analysis of agreement among journal referees. Scientometrics 19, 91–106 (1990).

[34] Lee C. J., A Kuhnian critique of psychometric research on peer review. Philos. Sci. 79, 859–870 (2012).

[35] H. Goldstein, *Multilevel Statistical Models* (John Wiley & Sons, 2011).

[36] S. W. Raudenbush, A. S. Bryk, *Hierarchical Linear Models: Applications and Data Analysis Methods* (SAGE, 2002).

[37] Benjamin D. J., Berger J. O., Johannesson M., Nosek B. A., Wagenmakers E. J., Berk R., Bollen K. A., Brembs B., Brown L., Camerer C., Cesarini D., Chambers C. D., Clyde M., Cook T. D., De Boeck P., Dienes Z., Dreber A., Easwaran K., Efferson C., Fehr E., Fidler F., Field A. P., Forster M., George E. I., Gonzalez R., Goodman S., Green E., Green D. P., Greenwald A. G., Hadfield J. D., Hedges L. V., Held L., Ho T. H., Hoijtink H., Hruschka D. J., Imai K., Imbens G., Ioannidis J. P. A., Jeon M., Jones J. H., Kirchler M., Laibson D., List J., Little R., Lupia A., Machery E., Maxwell S. E., Carthy M. M., Moore D. A., Morgan S. L., Munafó M., Nakagawa S., Nyhan B., Parker T. H., Pericchi L., Perugini M., Rouder J., Rousseau J., Savalei V., Schönbrodt F. D., Sellke T., Sinclair B., Tingley D., Van Zandt T., Vazire S., Watts D. J., Winship C., Wolpert R. L., Xie Y., Young C., Zinman J., Johnson V. E., Redefine statistical significance. Nat. Hum. Behav. 2, 6–10 (2018). 30980045 10.1038/s41562-017-0189-z

[38] Fleurence R. L., Forsythe L. P., Lauer M., Rotter J., Ioannidis J. P., Beal A., Frank L., Selby J. V., Engaging patients and stakeholders in research proposal review: The patient-centered outcomes research institute. Ann. Intern. Med. 161, 122–130 (2014). 25023251 10.7326/M13-2412

[39] Kahneman D., Klein G., Conditions for intuitive expertise: A failure to disagree. Am. Psychol. 64, 515–526 (2009). 19739881 10.1037/a0016755

[40] Higginson A. D., Munafò M. R., Current incentives for scientists lead to underpowered studies with erroneous conclusions. PLOS Biol. 14, e2000995 (2016). 27832072 10.1371/journal.pbio.2000995 PMC5104444

[41] Lindner M. D., Torralba K. D., Khan N. A., Scientific productivity: An exploratory study of metrics and incentives. PLOS ONE 13, e0195321 (2018). 29614101 10.1371/journal.pone.0195321 PMC5882165

[42] Smaldino P. E., McElreath R., The natural selection of bad science. R. Soc. Open Sci. 3, 160384 (2016). 27703703 10.1098/rsos.160384 PMC5043322

[43] Wang J., Veugelers R., Stephan P., Bias against novelty in science: A cautionary tale for users of bibliometric indicators. Res. Policy 46, 1416–1436 (2017).

[44] Warner E. T., Carapinha R., Weber G. M., Hill E. V., Reede J. Y., Faculty promotion and attrition: The importance of coauthor network reach at an academic medical center. J. Gen. Intern. Med. 31, 60–67 (2016). 26173540 10.1007/s11606-015-3463-7 PMC4700018

[45] Greenwald A. G., McGhee D. E., Schwartz J. L., Measuring individual differences in implicit cognition: The implicit association test. J. Pers. Soc. Psychol. 74, 1464–1480 (1998). 9654756 10.1037//0022-3514.74.6.1464

[46] Dovidio J. F., Gaertner S. L., Aversive racism and selection decisions: 1989 and 1999. Psychol. Sci. 11, 315–319 (2000). 11273391 10.1111/1467-9280.00262

[47] Blau F. D., Currie J. M., Croson R. T. A., Ginther D. K., Can mentoring help female assistant professors? Interim results from a randomized trial. Am. Econ. Rev. 100, 348–352 (2010).

[48] NIH Staff, *Definitions of Criteria and Considerations for Research Project Grant (RPG/R01/R03/R15/R21/R34) Critiques* (NIH, 2016).

[49] Hedges L. V., Effect sizes in cluster-randomized designs. J. Educ. Behav. Stat. 32, 341–370 (2007).

[50] R. J. A. Little, D. B. Rubin, *Statistical Analysis with Missing Data* (John Wiley & Sons, 2019).

[51] Hausman J. A., Specification tests in econometrics. Econometrica 46, 1251–1271 (1978).

