# Supplementary material for: NIH peer review: Criterion scores completely account for racial disparities in overall impact scores
Source: Sci Adv. 2020 Jun 3;6(23):eaaz4868. doi: 10.1126/sciadv.aaz4868 (PMC7269672; doi:10.1126/sciadv.aaz4868)
Supplement: aaz4868_SM.pdf [file aaz4868_SM.pdf]

Science Advances
AAAS

advances.sciencemag.org/cgi/content/full/6/23/eaaz4868/DC1

Supplementary Materials for

NIH peer review: Criterion scores completely account for racial disparities in overall impact scores

Elena A. Erosheva*, Sheridan Grant, Mei-Ching Chen, Mark D. Lindner, Richard K. Nakamura, Carole J. Lee*

*Corresponding author. Email: erosheva@uw.edu (E.A.E.); c3@uw.edu (C.J.L.)

Published 3 June 2020, *Sci. Adv.* **6**, eaaz4868 (2020)
DOI: 10.1126/sciadv.aaz4868

This PDF file includes:

Tables S1 to S10
Figs. S1 to S4
References

Supplementary Materials for: NIH Peer Review: Criterion Scores Completely Account for Racial Disparities in Overall Impact Scores

This project originated in 2014 when C.J.L. and E.A.E. won the Peer Review Challenge hosted by NIH and CSR, which led to a contract granting access to a limited confidential de-identified data set constructed from NIH records specifically for studying the relationship between preliminary criterion scores and preliminary overall impact scores at the level of individual reviewers. Specifically, we evaluate whether racial disparities in preliminary overall impact scores of assigned reviewers can be explained by other application and applicant characteristics and the hypothesized commensuration practices.

Study Data

This section provides a description of the study data, including key information from the main text for completeness. The data come from the NIH IMPAC II (Information for Management, Planning, Analysis, and Coordination) grant data system from the council years 2014-2016. This study focused on black-white disparities; we did not include 1,771 applications submitted by PIs whose race was American Indian or Alaskan, Asian, Native Hawaiian or Pacific Islander, or who indicated more than one race, as well as 8,648 applications for which PI race was withheld or unknown. Table 3 in the main text summarizes all variables used in our analyses and their definitions. At the time of application, PI demographics are voluntarily reported by applicants. CSR is not aware of patterns among PIs not reporting their demographic characteristics, though this issue deserves further attention if respondents who did not report these demographic characteristics are systematically different from others, which could lead to biased conclusions (*50*).

In the full set of 54,740 applications, approximately 15% of the applications from black and white PIs were missing information on PI gender, ethnicity (Hispanic/Latino or not), and degree, and were excluded from the study. Specifically, 232 were missing gender information, 7,409 were missing ethnicity information, and 1,639 were missing degree information. The remaining 46,226 applications—1,015 (or 2.2%) from black PIs and 45,211 (or 97.8%) from white PIs—were evaluated by 19,197 unique reviewers who wrote 139,216 reviews. 73.7% of the reviewers reviewed in just one of the three council years, 2014-2016, for which we have data, while 22.9% reviewed in two and 3.4% reviewed in three of those years. Because PIs can amend each application that is not funded initially and submit multiple applications for different projects, there are fewer unique PIs—500 (2.5%) black and 19,653 (97.5%) white. Among these applications with no missing data, there were 1,015 applications from 500 unique black PIs, which received 3,064 reviews from 2,322 unique reviewers (Table S1). There were 45,211 applications from 19,653 unique white PIs, which received 136,152 reviews from 19,100 unique reviewers. 73.7% of the reviewers reviewed for just one of the three council years during the period of 2014-2016, for which we have data, while 22.9% reviewed in two and 3.4% reviewed in three of those years.

Study codes—Human Subjects, Animal Subjects, Child, Gender, and Minority—are categorical variables that take on a number of values. For our analyses, all study codes were re-coded/coarsened to "Acceptable", "Unacceptable", or "Inapplicable", in order to avoid numerical estimation problems with rare categories and for ease of interpretability. Below, we describe only codes that occurred in our study data for example, code 20—no exemption

Table S1: Summary statistics for the FY 2014-2016 applicant pool.

Applicant Race	Applications	PIs	Reviewers	Review Records
Black	1,015	500	2,322	3,064
White	45,211	19,653	19,100	136,152
Total	46,226	20,153	19,197	139,216

designated, so award cannot be processed—never occurs in our data and is not discussed). Links to the current NIH study codes are provided as hyperlinked URLs in the text for ease of reference.

For *Human Subject codes* (https://www.niaid.nih.gov/grants-contracts/human-subjects-involvement-codes), code 10 (no human subjects involved) was re-coded to "Inapplicable," code 44 (human subjects involved, SRG concerns) to "Unacceptable," and other codes (30—certified with no SRG concerns; 54—previous concerns resolved; and exemptions) to "Acceptable." For Animal Subjects (https://www.niaid.nih.gov/grants-contracts/research-animals-involvement-codes), code 10 (no animal subjects involved) was re-coded to "Inapplicable," code 44 (animal subjects involved, SRG concerns) was re-coded to "Unacceptable," and others (30—animals involved with no SRG concerns; 32—animals involved with SRG comments; 48—conditional award with terms and conditions; 54—previous concerns resolved) to "Acceptable."

For *Gender codes* (https://www.niaid.nih.gov/grants-contracts/human-subjects-inclusion-codes), the categories of interest indicated whether or not women were knowingly included in the proposed study. Codes "1A" and "2A" were re-coded as "Acceptable," because they represent studies in which the researchers knowingly included women in the study design that were deemed acceptable. Codes "1U" and "2U" were re-coded as "Unacceptable," as they represent studies in which the researchers knowingly included women in the study design that were deemed unacceptable. The remaining applications—those whose proposed studies did not include human subjects, or did not knowingly include women—were re-coded as "Inapplicable."

The *Minority codes* (https://www.niaid.nih.gov/grants-contracts/human-subjects-inclusion-codes) and *Child codes* (the NIH Child Subjects codes for the data used in this study have since been updated to "Age codes" and can be found at https://grants.nih.gov/grants/funding/lifespan/review_codes.doc) are structured similarly to the Gender code and were re-coded analogously, with those applications in which minority subjects or child subjects were knowingly included being separated from the others in the re-coding. Note that, because the Human Subjects code indicates whether or not human subjects were included in the proposed study, the human subjects studies at the "Inapplicable" level of the Gender, Minority, and Child Subject codes are still recognized by our models as distinct from those without human subjects.

Finally, the *NIH Funding Bin* variable is determined by the amount of NIH R01 funding given to *all investigators* at an institution in 2014, and then split into 5 bins with roughly equal numbers of black PIs in each bin. These bins are delineated in Table S2.

Matching and Study Subsets Selection

While the observational units in our study are reviews, matching occurred at the application level. We used exact matching on eight key variables thought to be related to scores and

Table S2: Dollar award ranges for NIH funding bins.

Institution Bin	NIH Awarded Dollars Minimum	Maximum	Number of Black Awardees
1	$360,448,763	$593,400,359	200
2	$149,626,530	$360,448,762	203
3	$63,082,330	$149,626,529	206
4	$23,982,606	$63,082,329	194
5	$0	$23,982,605	212

award rates. Exact matching is a version of Coarsened Exact Matching (CEM) (*30*)—see proof in section Coarsened Exact Matching with Exact Matching on a Subset of Covariates. The matching variables, summarized in Table 1 of the main text, are: contact PI's gender, ethnicity, career stage, degree type, institution's NIH funding bin, application type, application's amended status (first submission or resubmission), and the area of science as represented by the Integrated Review Group (IRG).

CEM has several desirable properties including congruence (i.e., matching is performed on the data space rather than in a space of some metric such as the propensity score), relatively easy and flexible implementation, and Monotonic Imbalance Bounding (specifying the coarsening level for each variable automatically bounds imbalance allowed for each covariate) (*30*). It is recommended that coarsening levels be chosen based on subject matter knowledge about the measurement and the likely importance of different covariates (*30*). Due to the high number of categorical covariates, our choice was to carry out exact matching on eight key variables and implement complete coarsening for the rest; this choice achieved a good trade-off between improved balance and sample size. We tested coarsened exact matching on various additional covariates, but the ensuing reduction in sample size was prohibitive. Our matching procedure improved balance on all the matching variables and on most other applicant- and application-specific covariates (see Table S3). The improved balance makes estimates from the matched subset analysis more robust, or less susceptible to model misspecification, as compared to those from a random sample (*31-32*).

Matching Algorithm

This section relates the details of the matching algorithm, which was constructed to:

1. Maximize the number of applications in the matched data set;
2. Maximize the number of reviews of applications in the matched data set;
3. Enforce exact matching on the 8 matching variables (the remainder are "fully coarsened" and thus trivially matched in a CEM); and,
4. Respect the constraint that no more than four applications in the entire matched data set may come from the same reviewer. This constraint was implemented due to the sensitive nature of the data, in order to ensure the privacy and confidentiality of reviewers.

A near 1:2 matching was performed: each application from a black PI was matched with up to two applications from white PIs on the eight matching variables thought to be related to scores and/or award rates (see Table 1).

The selection of the matched data set was performed in two stages. First, black applications were matched to white applications to form a "matched tuple" of 1 black and either 1 or 2 white applications. Then, review records were selected for each set of matched applications using the following algorithm:

1. Create a dictionary in which the keys are reviewers and the values are the number of reviews from the given reviewer that have been selected. Initialize all values to zero. If a reviewer's value is less than five, that reviewer's reviews are "eligible"; five or more, and they are ineligible.

2. For each matched tuple:

   (a) For each application in the matched tuple:

      i. If any reviews of the application are eligible, select one at random, add it to the matched data set, and add one to the value of the appropriate reviewer key.

      ii. If no reviews of the applications are eligible:

         A. If the application is from a black applicant, discard the application and the associated matched white application(s).

         B. If the application is from a white applicant, discard the application and replace it with another exact match from among the white applications, if available.

3. Repeat step 2 until there are no remaining eligible reviews to be selected.

This algorithm attempts to maximize the number of records in the data set by minimizing the number of matched tuples/black applications that must be discarded. It does this by selecting only one review for as many applications as possible before selecting the remaining eligible reviews. No black applications and nine white applications were discarded by this algorithm when the final study data were selected.

Random Subset Selection

The random subset selection algorithm was designed to generate as representative a set of reviews of white applicants as possible while respecting the constraint that no more than four reviews from a given reviewer may be in the data set. Its steps are as follows:

1. Randomly select twice the number of available black applications from the full set of white applications (for this data set, there were 1,015 black applications and so 2,030 randomly selected white applications were included). Call this number n (here $n = 2,030$).

2. For each application, select one eligible review, or discard the application if no reviews are eligible. Repeat until there are no remaining eligible reviews.

3. While the number of applications in the random sample is less than n, randomly select an application from the set of white applications in which at least one reviewer of each application reviewed 5 or more applications, and in which each application has at least one eligible review. Add one to the value associated with the appropriate reviewer key of the dictionary described in step 1 of the matching algorithm. Select reviews from this application as in step 2a of the matching algorithm.

A naive approach to random subset selection would replace each application with no eligible reviewers with another randomly selected application. However, this would systematically bias the sample to include applications that were reviewed by less experienced reviewers at a higher rate than in the population. By replacing applications with no eligible reviews with applications that were also reviewed by at least one experienced reviewer (a reviewer with 5 or more reviews in the data set), we mitigate this bias.

Coarsened Exact Matching with Exact Matching on a Subset of Covariates

In this section, we prove that exact matching on selected variables is a version of Coarsened Exact Matching (CEM). In this section only, we use the language of potential outcomes and treatment effects for the ease of exposition and to reflect the language used in (*30*). We emphasize that although our analysis relies on matching, it reports conditional associations and it does not present estimates of causal effects.

Exact matching on a strict subset of the covariates is CEM with "full coarsening" on the unmatched covariates. One may verify this by checking that the proofs in (*30*) do not assume that each coarsened variable has at least 2 coarsened levels. We now provide an in-depth example proof for the boundedness of SATT (Sample Average Treatment Effect on the Treated) estimation error.

Equation (7) of (*30*) states that as long as the true treatment effect is a Lipschitz function of the observed covariates and the maximum width of a coarsening interval (or set, for categorical variables) is $\epsilon_j < \infty$ for the j-th covariate, then the SATT absolute estimation error is bounded. For this to hold with exact matching on a covariate subset, we simply require that the range of each non-matching variable be bounded by some ϵ_j for an appropriate metric in addition to the Lipschitz requirement. Bounded range is equivalent to having a bounded coarsening interval width, which is nearly always the case in practice; furthermore, if no such ϵ_j exists then no coarsening will yield finite ϵ_j and thus the requirement is not restrictive. Explicitly, for continuous non-matching variables we require the range to be finite, for ordinal non-matching variables we require a finite number of levels, and for categorical non-matching variables we impose no restriction as the distance between any two levels of a categorical variable can be said to be one. For the exact matching variables, we have $\epsilon_j = 0$ by definition. These assumptions do not impose much in practice. Let $X_1, \ldots, X_k$ be the observed variables with $X_{i1}, \ldots, X_{ik}$ the values for the i-th observation. Then let the potential outcome at treatment level 0 be $Y_i(0)$ (in this paper, treatment level zero corresponds to being white) for the i-th individual. Under the ignorability assumption, we can write

$$Y_i(0) = g_0(X_{i1}, \ldots, X_{ik})$$

where we have omitted possible mean-zero noise for ease of exposition (this is justifiable because ignorability guarantees that this noise is independent, so it does not contribute any bias to our estimator; it only adds to its variability). Since we always observe $Y_i(1)$ for treated individuals, our estimate of the treatment effect for the treated is

$$\widehat{TE}_i = Y_i(1) - \widehat{Y_1(0)}$$

and the true treatment effect for the treated is

$$TE_i = Y_i(1) - Y_i(0).$$

For the difference-in-means estimator, $\widehat{Y_i(0)}$ is also the observed outcome for a matched untreated unit. We then have

$$\begin{aligned} TE_i &= \widehat{TE}_i + \widehat{Y_i(0)} - Y_i(0) \\ &= \widehat{TE}_i + g_0(\widetilde{X}_1) - g_0(X_i) \end{aligned}$$

where $\tilde{X}_i$ represents the covariate vector for the observation matched to the i-th treated unit, with observed outcome $\widehat{Y_i(0)}$. Taking an average over the treated units, we get

$$SATT = \widehat{SATT} + \frac{1}{n_T} \sum_{i=1}^{n_T} g_0(\tilde{X}_i) - g_0(X_i).$$

Now, assume that g_0 is Lipschitz in the sense that replacing X_j with any $\tilde{X}_j$ within the coarsened matching caliper of width ϵ_j and holding all other variables fixed changes the value of g_0 by at most L_j for any j. Then for any i,

$$|g_0(\widetilde{X}_t) - g_0(X_i)| \leq \sum_j L_j \epsilon_j$$

and, as desired, it immediately follows that

$$|SATT - \widehat{SATT}| \leq \sum_j L_j \epsilon_j.$$

For another example, consider Section 4.1 of (*30*), regarding the maximum imbalance bound. For the non-matching variables, the imbalance bound is simply 1 (although in practice the imbalance is typically much less than 1, as can be seen from Table S3), and it remains true that specifying a coarsening for one variable does not affect the imbalance bound for other variables because the maximum possible imbalance under the L_1 distance is 1. As noted by (*30*), this property stands in contrast to certain Mahalanobis distance matching methods where the user demands a certain sample size from the matching, in which additionally imposing an upper bound on balance or coarsening for one variable can increase the imbalance bound for other variables.

Balance Analysis

The goal of matching is to increase balance between treated and untreated units. After matching, we check balance on the application- and applicant-specific covariates between black and white applicants. Our measure of balance is L1 overlap, or one minus the total variation distance. Rather than simply assessing differences in means and standard deviations by covariate, the L1 overlap measures how different the entire empirical distributions of the variables are between the black and white subsets. The L1 approach to measuring overlap—recommended by (*30*)—is superior because the entire distribution of a covariate is of concern when a model is misspecified, and model misspecification is one of the main concerns matching is designed to address.

Table S3 displays the L1 overlap (on a zero-to-one scale) for the random and matched subsets, as well as the percentage increase in overlap for the matched subset. Exact matching variables are in bold. Note that overlap for exact matched variables may not be exactly 1 because the matching is not strictly one-to-one, so the distributions in the white and black

Table S3: L1 overlap for control covariates; exact matching variables are in bold.

	L1 Overlap		
Variable	Random	Matched	Increase
Institute/Center	0.83	0.90	8%
**IRG**	0.77	0.99	28%
SRG	0.89	0.91	2%
Institution Sector	0.95	0.94	-1%
Graduate Education	1.00	1.00	0%
IPEDS Lookup	0.98	0.96	-2%
MSI Type (council year 2015)	0.97	0.97	0%
Solicitation Type	0.97	0.99	1%
**Amended Status**	0.95	1.00	5%
Multiple PIs	0.98	0.99	1%
Support Years	0.96	0.99	2%
Council Year	0.96	0.96	0%
Review Group Type	0.94	0.94	0%
**Application Type**	0.90	1.00	11%
Human Subjects	0.76	0.95	24%
Animal Subjects	0.85	0.99	16%
Gender Code	0.90	0.98	9%
Minority Code	0.90	0.99	9%
Child Code	0.90	0.98	9%
**Gender**	0.91	1.00	10%
**Ethnicity**	0.98	1.00	2%
**Degree Type**	0.88	0.98	11%
Terminal Degree Year	0.78	0.85	9%
NIH Funding History	0.89	0.97	9%
**Career Stage**	0.76	0.99	31%
**NIH Funding Bin**	0.93	0.99	6%
Geographic Location	0.87	0.89	2%
(Log) Requested Costs	0.83	0.91	9%

matched subsets may differ slightly. Overall, the overlap noticeably improved for the 8 exact matching covariates, and improved moderately for most other covariates. Note that the overlap for Institution Sector and Institution Lookup variables declined slightly after matching. While this is not a major concern because the overlap is still quite high, we note that CEM does not guarantee that imbalance will improve on every covariate, merely that there is an upper bound on imbalance for each covariate.

Multilevel Modeling

This section provides a description of the multilevel (hierarchical) linear models, starting with the model equations given in the Materials and Methods section of the main paper. We specified linear models for preliminary overall impact scores at the review level, relying on the NIH review structure and distinguishing between "structural variables" and other covariates that could potentially be associated with preliminary overall impact scores. IRG, SRG, and administering institute as well as reviewer and PI indicators are structural variables as they represent various levels of clustering in the data. The structure, as illustrated in Figure 1 of the main paper, is as follows. Reviews are clustered in a mixed hierarchy: reviews are nested within applications, which are nested within PIs. But reviewers can review multiple PIs just as PIs are reviewed by multiple reviewers: reviewer and PI are "crossed." Applications are nested within SRG, IRG, and administering institute, but PIs are not: over 200 PIs had applications reviewed in more than one SRG, IRG, or administering institute. All SRGs are nested within IRG, and IRG and SRG are both crossed with administering institute. All special emphasis panels within an IRG were modeled as a single study section.

All of our models account for structural dependencies in the data via random and fixed effects for the structural variables. Random intercepts are appropriate when the observed values of a clustering variable can be regarded as a sample from some larger population, whereas fixed effects are appropriate when the levels of a clustering variable are considered fixed and known. To this end, we model fixed effects for IRG and administering institute, because these are large, well-established units that are unlikely to change over time. We fit random intercepts for SRGs, because SRGs are created and disbanded routinely (and as a result of a Hausman test, discussed subsequently). PIs, applications, and reviewers all merit random effects because the observed values of these variables are samples from larger populations of PIs, applications, and reviewers. Application random intercepts were excluded from the models as they were redundant (i.e., estimated to have zero variability) after estimating PI random intercepts.

Model Specifications

Let Y_{ijklm} be the preliminary overall impact score for the ith review of the jth application from the kth PI (reviewed by the lth reviewer in the mth SRG), R_k a race indicator (1 indicates a black PI), and X_{jk} a vector of application- and applicant-specific control variables. To estimate racial disparities, we consider the following mixed effects model formulation:

$$Y_{ijklm} = \alpha + \beta_R R_k + \beta X_{jk} + \gamma_k + \xi_l + \eta_m + \epsilon_{ij}$$

where α is the model intercept; β_R is the race coefficient; β is the vector of coefficients for control variables; γ_k, ξ_l, and η_m are random intercepts for PI, reviewer, and SRG; and the ϵ_{ij} are within-application independent Gaussian error terms. Here, only SRG could have potentially been specified as a fixed effect. Conducting the Hausman test (*51*) for specification of the SRG effects, we conclude that the random effects specification is plausible; additionally, it aligns well with our substantive knowledge that SRGs are occasionally disbanded or created over time and can thus be thought of as coming from a hypothetical "population of SRGs." We examine estimates of the race coefficient β_R from a series of models: first only adjusting for the structural covariates and then including applicant- and application-level characteristics and preliminary criterion scores among the control covariates X.

To study commensuration practices, we focus on interaction effects between race and the preliminary criterion scores. Let Z_{ij} be the vector of criterion scores associated with the ith review of the jth application. The linear commensuration model for the preliminary overall impact score Y_{ijklm} of the ith review of the jth application from the kth PI (reviewed by the lth reviewer in the mth SRG) is specified by

$$Y_{ijklm} = \alpha + \beta_R R_k + \beta_C Z_{ij} + \beta_I R_k Z_{ij} + \beta X_{jk} + \gamma_k + \xi_l + \eta_m + \epsilon_{ij}$$

where α is the model intercept; β_R is the race coefficient; β_C is a vector of preliminary criterion score coefficients; β_I is the vector of coefficients for the interactions between race and the preliminary criterion scores ("commensuration coefficients"); β is the vector of coefficients for control variables X_{jk}; γ_k, ξ_l, and η_m are random intercepts for PIs, reviewers, and SRGs; and the ϵ_{ij} are within-application independent Gaussian error terms. For commensuration models, the control variables X include structural and applicant- and application-level characteristics. Note that because the focus of our analyses is on the relationship between preliminary criterion and preliminary overall impact scores, and because we do not have a reason to think that the reviewers' consideration of preliminary criterion scores in assigning the preliminary overall impact score would be different depending on application type (Type 1 or Type 2, new submission or resubmission), we use data for all application types in our models and control for application types with fixed effects.

Hausman Test for SRG Effects

Our models may account for clustering by SRG using either fixed or random effects. Random effects have the benefit of aligning with our substantive knowledge that SRGs at NIH are not always fixed but may appear or disappear over time; random effects models use fewer degrees of freedom; and, if the assumption of no endogeneity holds in that there is no correlation between the random intercepts and model residuals, fixed effects estimates from random effects models are asymptotically efficient. However, if endogeneity is present, the random effect model estimates for the fixed effects (the coefficients of interest in this paper) are inconsistent while those of the fixed effect model are still consistent.

The null hypothesis for the Hausman test is that the fixed-effects coefficients are consistent in both the SRG random effects and SRG fixed effects models, and consequently that the SRG random effect model estimates are efficient. It is shown in (*51*) that the covariance between an asymptotically efficient estimator and its difference from a different consistent but inefficient estimator is asymptotically zero. A simple chi-squared test can be constructed based on this result with the following statistic and null distribution

$$\left(\widehat{\beta_{RE}} - \widehat{\beta_{FE}}\right)^T \left(\text{Cov}\left(\widehat{\beta_{RB}}\right) - \text{Cov}\left(\widehat{\beta_{FB}}\right)\right)^{-1} \left(\widehat{\beta_{RB}} - \widehat{\beta_{FB}}\right) \sim \chi^2_p$$

where RE stands for random effects and FE for fixed effects, p is the number of fixed-effect coefficients estimated in the model (and the number of degrees of freedom of the chi-square distribution), and the inverse is a pseudo-inverse. Because under local alternatives to the null (i.e. slight model misspecification) the test statistic has a noncentral chi-square distribution (i.e. larger expected value), we reject the null if the test statistic is too large. For the matched subset analysis of the full data set (the analysis presented in the main text), this statistic was 28.76 on 115 degrees of freedom, with p-value approximately 1. The test fails to reject the null hypothesis of no endogeneity, and since random effects align well with our substantive knowledge of the SRG, we elect to fit SRG random effects. In practice, both

random effects and fixed effects models lead to the same substantive conclusions and very similar coefficient estimates for our data.

Model Diagnostics

We assess the fit of our commensuration model (from the matched subset analysis) to the data to ensure that hierarchical linear mixed-effects model assumptions are satisfied. For mixed-effects models, residual analysis constitutes the bulk of the diagnostics. There are three main types of residuals in mixed-effects models: conditional residuals, marginal residuals, and BLUPs (best linear unbiased predictors). If y is the outcome, x the observed covariates, $\hat{\beta}$ the estimated fixed-effect coefficients, and $\hat{\gamma}$ the best linear unbiased predictor of the random effects, then:

- the conditional residuals are $e_c = y - \hat{\beta}x - \hat{\gamma}$,
- the marginal residuals are $e_m = y - \hat{\beta}x$, and
- the BLUPs are $\hat{\gamma} = e_m - e_c$.

Any one of these residuals can be computed from the other two; hence examination of only two types of residuals is needed. For our analyses, we examined normal quantile-quantile plots for the conditional residuals and BLUPs for the three random intercepts included in the commensuration model (matched subset analysis) (the plots are not shown). The conditional residuals and BLUPs displayed wider tails than a normal distribution, indicating some excess kurtosis that was not enough to raise any concerns given the large sample size and robustness of linear regression to deviations from normality of residuals. Furthermore, no conditional residuals nor BLUPs displayed evidence of heteroscedasticity or dependence on the main covariates of interest (i.e., race, the preliminary criterion scores, requested costs—the only purely continuous covariate—and terminal degree year, the only ordinal covariate aside from the preliminary criterion scores). For both residual types, residual analysis plots indicated that assumptions of homoscedasticity, independence between residuals and covariates, and approximate Gaussianity are reasonable and that our model estimates are valid under the proposed model class of hierarchical linear mixed-effects models.

Commensuration Practices

To interpret the magnitude of the estimated commensuration coefficients, we examine estimated expected differences in scores for black versus white applications (Figure S1) and a hypothetical example of differences in predicted preliminary overall impact scores for given preliminary criterion scores between black and white applications (Table S4).

Figure S1 shows the expected change in preliminary overall impact score for all black applications if their criterion scores were commensurated into overall impact scores as if these were applications submitted by white PIs with otherwise identical values for the observed application- and applicant-specific covariates. For just 15% of black applications would we expect an otherwise identical (on the observed covariates) white application to score differently by at least 0.1 points (better, 11%; worse, 4%) in the preliminary overall impact score due to commensuration differences. The difference of 0.1 points in preliminary overall impact score is not large relative to the variability due to other sources. As explained in the main paper, a change in an application's overall impact score of 0.3 points is substantial because it could tangibly affect funding decisions. At the same time, we point

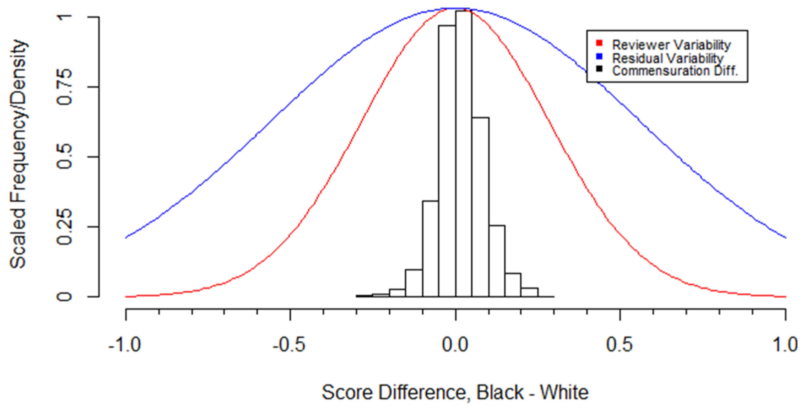

Figure S1: Distribution of estimated expected preliminary overall impact score differences due to commensuration (histogram) and distributions of reviewer intercepts and model residuals (colored lines), under the **matched subset commensuration model** (Table 6). Histogram and densities have been scaled to have a common maximum for ease of visualizing differences in variability.

out that 0.3 points is similar in magnitude to the (estimated standard deviation due to) reviewer variability, and is about twice as small as the estimated residual standard deviation after controlling for preliminary criterion scores (Models 3 and 4, Table 5). Based on the commensuration model for the matched subset, 15% of all applications can expect the preliminary overall impact score to differ by at least 0.1 points from the average score due to random reviewer variability, and 86% due to residual variability that is not explained by the model. No black application would expect to see a score difference of greater than 0.3 because of commensuration practices as estimated on the matched subset (Figure S1). The population-weighted expected average difference in preliminary overall impact score between black and white applications, conditional on the control covariates, is 0.004, which is practically negligible. This quantity, 0.004, is small because the interaction coefficient estimates are balanced between positive and negative quantities and all the preliminary criterion scores are positively correlated. Combined, these two facts lead to a "cancelling" phenomenon in which the estimated expected difference in preliminary overall impact score is small for the vast majority of applicants.

In addition, to illustrate the potential impact of commensuration differences on the preliminary overall impact score, we consider two situational pairs, each of a hypothetical black applicant and a hypothetical white applicant (Table S4). Here, we pick scenarios where the discrepancies between preliminary Approach and Significance/Innovation scores are extreme but still plausible: each combination of criterion scores does occur in our dataset both for white and black applicants. The "Innovative" preliminary criterion score combination occurs twice in the set of reviews of black applications and once in the set of reviews of white applications; the "Thorough" preliminary criterion score combination occurs twice in the set of white applicants and twice in the set of black applicants. In each hypothetical scenario, we assume the two applicants and applications are identical on all the observed covariates except for race.

Under the "Innovative" scenario, the application review scores indicate that the proposed

Table S4: Hypothetical Commensuration Scenarios

	Innovative		Thorough	
Applicant Race	White	Black	White	Black
Significance	1	1	5	5
Investigator	2	2	2	2
Innovation	2	2	5	5
Approach	5	5	2	2
Environment	2	2	2	2

Hypothetical preliminary criterion score scenarios; "innovative" scenario has relatively high Innovation and Significance scores and a low Approach score, and vice-versa for the "thorough" scenario.

research is innovative and significant, but that the approach is sub-par. Based on our matched subset commensuration analysis, in such a scenario, the matched white researcher's score would be 0.12 points better than the black researcher's score ($p < 0.005$). This difference occurs because, on average, reviewers weigh the preliminary Approach score more heavily for black applicants than for matched whites and the preliminary Innovation and Significance scores less heavily. Conversely, under the "Thorough" scenario, in which the research proposals are scored as rigorous but not significant or innovative, our model predicts that the black applicant will, on average, receive an impact score 0.20 points better than the white researcher ($p < 0.005$). As noted earlier, these differences are small in magnitude as compared to reviewer random effect variability or residual variability.

Random Subset Analyses

While we emphasize the results of our matched subset analyses as less susceptible to model specification, we also performed random subset analyses in the interests of comparison and to allay concerns that our matching design was suboptimal. The main results of these analyses are presented alongside the matched subset analyses below in Tables S5 and S6.

The conclusions one draws from the random subset analysis are largely the same as those of the matched subset analysis, given the very similar coefficient estimates and significance levels. One point of difference is that the race coefficient in the random subset commensuration analysis is statistically significant, while it is not for the matched subset analysis. However, because the commensuration model includes interaction coefficients between PI race and the criterion scores, this coefficient cannot be interpreted on its own. Figure S2 shows the expected change between black and white applications due to commensuration practices as estimated with the random subset analysis. As with our main analysis (Figure S1), estimated expected differences in preliminary overall impact score of 0.1 points or more as a result of commensuration differences are rare. The population-weighted expected average difference in preliminary overall impact score between black and white applications, conditional on the control covariates, is 0.038 (in favor of white applications) under the random subset analysis which is similar to that of 0.004 under the matched subset analysis. These expected changes in preliminary overall impact scores due to commensuration practices are practically negligible.

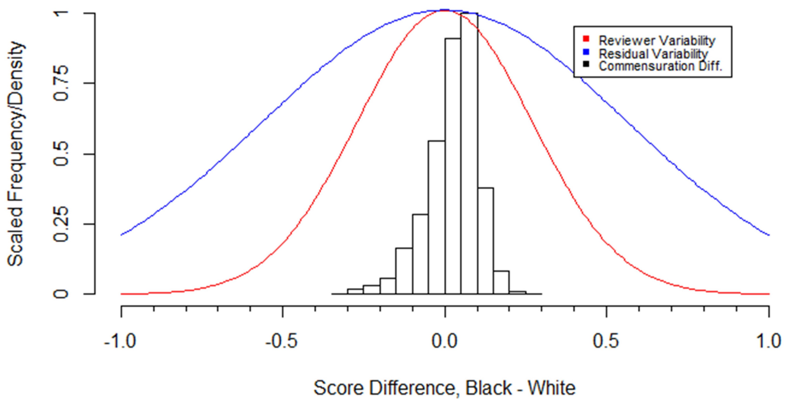

Figure S2: Distribution of estimated expected preliminary overall impact score differences due to commensuration (histogram) and distributions of reviewer intercepts and model residuals (colored lines), under the **random subset commensuration model** (Table S6). Histogram and densities have been scaled to have a common maximum for ease of visualizing differences in variability.

Table S5: Selected parameter estimates from Models 1-4, matched and random subsets.

Subset	Parameters	Model 1	Model 2	Model 3	Model 4
Matched	*Race Fixed Effect*				
	Coefficient	0.466*	0.350*	0.010	0.014
	(Std. Err.)	(0.062)	(0.051)	(0.017)	(0.018)
	p-value	< 0.005	< 0.005	0.561	0.431
	Effect Size	0.358	0.272	0.018	0.025
	Criterion Score Fixed Effects				
	Significance (Std. Err.)			0.250 (0.007)	0.245 (0.007)
	Investigator (Std. Err.)			0.068 (0.008)	0.064 (0.009)
	Innovation (Std. Err.)			0.125 (0.007)	0.122 (0.007)
	Approach (Std. Err.)			0.625 (0.006)	0.612 (0.006)
	Environment (Std. Err.)			0.018 (0.009)	0.017 (0.009)
	Random Effects				
	Reviewer Std. Dev.	0.507	0.500	0.286	0.286
	PI Std. Dev.	0.883	0.578	0.100	0.082
	SRG Std. Dev.	0.343	0.271	0.084	0.074
	Residual Std. Dev.	1.300	1.284	0.566	0.562
Random	*Race Fixed Effect*				
	Coefficient	0.700*	0.418*	0.031	0.031
	(Std. Err.)	(0.064)	(0.056)	(0.017)	(0.018)
	p-value	< 0.005	< 0.005	0.071	0.078
	Effect Size	0.533	0.322	0.054	0.055
	Criterion Score Fixed Effects				
	Significance (Std. Err.)			0.251 (0.006)	0.246 (0.006)
	Investigator (Std. Err.)			0.089 (0.008)	0.085 (0.008)
	Innovation (Std. Err.)			0.128 (0.006)	0.127 (0.006)
	Approach (Std. Err.)			0.638 (0.005)	0.623 (0.005)
	Environment (Std. Err.)			0.010 (0.009)	0.005 (0.009)
	Random Effects				
	Reviewer Std. Dev.	0.490	0.502	0.274	0.272
	PI Std. Dev.	0.836	0.651	0.093	0.080
	SRG Std. Dev.	0.306	0.256	0.084	0.066
	Residual Std. Dev.	1.312	1.296	0.567	0.564

Race coefficient estimates and their effect sizes; preliminary criterion score fixed effects and their standard errors; and variance components estimates from four hierarchical linear models for preliminary overall impact scores fit on $n = 7471$ reviews of 2566 applications (matched subset) and $n = 8595$ reviews of 3045 applications (random subset). Model 1 controls for structural covariates; Model 2 controls for structural and applicant-/application-specific covariates; Model 3 controls for structural covariates and preliminary criterion scores; Model 4 controls for structural, applicant/application-specific covariates, and preliminary criterion scores. Control variables are listed in Table 3 of the main paper. Coefficient estimates for control variables are not shown. Significance * is reported for the race fixed effect estimate for $p < .005$. In mixed effects models, multiple effect sizes exist for a given coefficient; we report the coefficient divided by the residual standard deviation. For more information, see (*49*).

Table S6: Selected parameter estimates, commensuration models.

Variable	Matched Subset Estimate (Std. Err.)	Matched Subset P-Val.	Random Subset Estimate (Std. Err.)	Random Subset P-Val.
Fixed Effects				
Significance	0.258 (0.008)*	< 0.005	0.256 (0.008)*	< 0.005
Investigator	0.057 (0.011)*	< 0.005	0.098 (0.011)*	< 0.005
Innovation	0.129 (0.008)*	< 0.005	0.141 (0.008)*	< 0.005
Approach	0.598 (0.007)*	< 0.005	0.613 (0.007)*	< 0.005
Environment	0.022 (0.011)	0.057	0.000 (0.012)	0.992
PI Race = Black	-0.024 (0.047)	0.610	0.139 (0.044)*	< 0.005
Significance * PI Black	-0.034 (0.013)	0.010	-0.023 (0.012)	0.062
Investigator * PI Black	0.018 (0.017)	0.298	-0.027 (0.017)	0.105
Innovation * PI Black	-0.021 (0.014)	0.144	-0.038 (0.013)*	< 0.005
Approach * PI Black	0.041 (0.012)*	< 0.005	0.026 (0.011)	0.021
Environment * PI Black	-0.010 (0.018)	0.596	0.016 (0.018)	0.373
Random Effects				
Reviewer Intercepts Std. Dev.	0.286		0.272	
PI Intercepts Std. Dev.	0.079		0.078	
SRG Intercepts Std. Dev.	0.076		0.066	
Residual Variability Std. Dev.	0.562		0.564	

Preliminary criterion, race, commensuration (race-criterion interaction) coefficients and variance components estimates for preliminary overall impact scores on $n = 7471$ reviews of 2566 applications (matched subset) and $n = 8595$ reviews of 3045 applications (random subset). Coefficient estimates for control variables that include structural and applicant-/application-specific covariates as listed in Table 3 of the main paper are not shown. Significance * is reported for $p < .005$.

Post-Discussion Scores

This analysis is for applications that have reached the SRG discussion stage. Not all reviewers change their criterion and overall impact scores after discussion (Table S7). Among post-discussion reviews, 20% saw a change in both the overall impact score and at least one criterion score post-discussion, 27% saw a change in the overall impact score but not in the criterion scores, 4% saw a change in the criterion scores but not in the overall impact score, and 49% saw no change in any scores. Thus only 20% of reviewers had revised both the criterion scores and the overall impact score based on the discussion (sometimes, a reviewer will decide that their assessment remains correct after the discussion, or will decide that changes to criterion scores do not merit a change in the overall impact score).

Table S7: Percentage of Reviews with Scores Changed

	Criterion Scores Changed	Criterion Scores Unchanged
Impact Score Changed	0.20	0.27
Impact Score Not Changed	0.04	0.49

Score change behavior after discussion; discussed reviews only.

With the caveats as stated above, we replicate the racial disparity analysis for final overall impact scores (Table S8). The results are largely the same: controlling for final criterion scores accounts for essentially all racial disparities in final overall impact scores. Note, though, that the racial disparities observed without controlling for final criterion scores are not as large as those for preliminary scores.

Table S8: Selected parameter estimates from Models 1-4, matched and random subsets.

Subset	Parameters	Model 1	Model 2	Model 3	Model 4
Matched	*Race Fixed Effect*				
	Coefficient	0.172	0.131	0.047	0.044
	(Std. Err.)	(0.082)	(0.080)	(0.043)	(0.045)
	p-value	0.035	0.101	0.276	0.329
	Effect Size	0.180	0.143	0.077	0.073
	Random Effects				
	Reviewer Std. Dev.	0.144	0.184	0.170	0.190
	PI Std. Dev.	0.954	0.890	0.470	0.471
	SRG Std. Dev.	0.396	0.366	0.154	0.169
	Residual Std. Dev.	0.942	0.917	0.613	0.595
Random	*Race Fixed Effect*				
	Coefficient	0.458*	0.261*	0.163	0.114
	(Std. Err.)	(0.079)	(0.078)	(0.043)	(0.045)
	p-value	< 0.005	< 0.005	< 0.005	0.011
	Effect Size	0.500	0.296	0.280	0.199
	Random Effects				
	Reviewer Std. Dev.	0.247	0.276	0.178	0.186
	PI Std. Dev.	0.937	0.847	0.471	0.464
	SRG Std. Dev.	0.370	0.383	0.185	0.210
	Residual Std. Dev.	0.907	0.883	0.586	0.573

Race coefficient estimates, their effect sizes, and variance components estimates from four hierarchical linear models for **final** overall impact scores fit on $n = 3921$ reviews (matched subset) and $n = 4543$ reviews (random subset). Model 1 controls for structural covariates; Model 2 controls for structural and matching covariates; Model 3 controls for structural covariates and criterion scores; Model 4 controls for structural, matching covariates, and criterion scores. Coefficient estimates for control variables are not shown. Significance * is reported for $p < .005$.

Reproducibility

Because of the sensitive nature of individual-level data, a reduced data set that contains the same reviews but fewer covariates is available for public use. This public-use data set includes all of the covariates of interest (applicant race, preliminary criterion and overall impact scores), the structural covariates (PI ID, application ID, reviewer ID, administering institute ID, IRG ID, and SRG ID), the matching variables (contact PI's gender, ethnicity, career stage, degree type, institution's NIH funding bin, application type, application's amended status, and the area of science represented by the Integrated Review Group), as well as the final overall impact score. We provide the url of the public-use data depository in the Acknowledgements section of the main text.

Here, we reproduce results of the multilevel analysis of racial disparities in preliminary overall impact scores from Table 5 and of commensuration practices in Table 6, for the matched and random subsets, using the public use data set. We also reproduce Figures S1 and S2 of the expected change in preliminary overall impact score for all black applicants if their preliminary criterion scores were commensurated into preliminary overall impact scores as if they were white, for the matched subset of the public-use reduced-covariates data set.

Racial disparities: Table S9 presents multilevel modeling results from the public-use data that are analogous to those reported in Tables 5 and S5. We find that the race coefficient estimates from Models 1 and 2 (which do not control for preliminary criterion scores) obtained from public-use data are positive, statistically significant, and very similar in magnitude to those reported in Tables 5 and S5. Once preliminary criterion scores are included (Models 3 and 4), the race coefficient estimates obtained from the matched and random subsets of the public-use data set (Table S9) become practically and statistically insignificant, similarly to our results from the matched (Table 5) and random (Table S5) subsets of the confidential data set. These results are consistent with our respective interpretation of racial disparities in the main paper. While the main results concerning the race coefficient estimates are strikingly similar between the confidential and the public use data set, we note that the random intercept variability for PIs and SRGs is somewhat larger for Model 2 fit to the matched subset of the public-use data set (Table S9) than for Model 2 fit to matched subset of the confidential study data set (Table 5). This is because fewer PI-specific covariates are included in the model as there are fewer covariates available in the public data set to explain PI variability.

Commensuration practices: Table S10 contains relevant parameter estimates from the linear commensuration models that were fit using the public data for both the matched and random white subsets. For the matched subset, which is less susceptible to model misspecification (*31-32*), the signs and magnitude of coefficient estimates for the interaction coefficients are strikingly similar to our main results on commensuration practices from Table 6. For the random subset, we note that the pattern of significant commensuration practices coefficients changes slightly (compare with Table S6). However, as Figures S3 and S4 demonstrate, the combined extent and magnitude of commensuration differences across all preliminary criterion scores remains small: expected differences for black applications in the preliminary overall impact score of 0.1 or more as result of commensuration practices are rare. This finding is consistent across all our analyses—random/matched subsets of confidential and reduced-covariate public-use data sets.

Table S9: Selected parameter estimates from Models 1-4, matched and random subsets.

Subset	Parameters	Model 1	Model 2	Model 3	Model 4
Matched	*Race Fixed Effect*				
	Coefficient	0.466*	0.431*	0.010	0.014
	(Std. Err.)	(0.062)	(0.057)	(0.017)	(0.017)
	p-value	< 0.005	< 0.005	0.561	0.412
	Effect Size	0.358	0.333	0.018	0.025
	Random Effects				
	Reviewer Std. Dev.	0.507	0.501	0.286	0.288
	PI Std. Dev.	0.883	0.769	0.100	0.097
	SRG Std. Dev.	0.343	0.304	0.084	0.087
	Residual Std. Dev.	1.300	1.296	0.565	0.563
Random	*Race Fixed Effect*				
	Coefficient	0.700*	0.497*	0.031	0.026
	(Std. Err.)	(0.064)	(0.060)	(0.017)	(0.017)
	p-value	< 0.005	< 0.005	0.071	0.143
	Effect Size	0.533	0.382	0.054	0.045
	Random Effects				
	Reviewer Std. Dev.	0.490	0.509	0.274	0.275
	PI Std. Dev.	0.836	0.803	0.093	0.090
	SRG Std. Dev.	0.306	0.289	0.084	0.085
	Residual Std. Dev.	1.312	1.302	0.567	0.565

Public-use data set: Race coefficient estimates, their effect sizes, and variance components estimates from four hierarchical linear models for preliminary overall impact scores fit on $n = 7471$ reviews of 2566 applications (matched subset) and $n = 8595$ reviews of 3045 applications (random subset). Model 1 controls for structural covariates; Model 2 controls for structural and matching covariates; Model 3 controls for structural covariates and criterion scores; Model 4 controls for structural, matching covariates, and criterion scores. Coefficient estimates for control variables are not shown. Significance * is reported for $p < .005$.

Table S10: Selected parameter estimates, commensuration model.

Variable	Matched Subset Estimate (Std. Err.)	Matched Subset P-Val.	Random Subset Estimate (Std. Err.)	Random Subset P-Val.
Fixed Effects				
Significance	0.263 (0.008)*	< 0.005	0.259 (0.008)*	< 0.005
Investigator	0.060 (0.011)*	< 0.005	0.099 (0.011)*	< 0.005
Innovation	0.132 (0.008)*	< 0.005	0.143 (0.008)*	< 0.005
Approach	0.604 (0.007)*	< 0.005	0.618 (0.007)*	< 0.005
Environment	0.019 (0.011)	0.090	0.002 (0.012)	0.841
PI Race = Black	-0.031 (0.047)	0.508	0.125 (0.044)*	< 0.005
Significance * PI Black	-0.035 (0.012)	0.008	-0.021 (0.013)	0.097
Investigator * PI Black	0.017 (0.017)	0.337	-0.030 (0.017)	0.073
Innovation * PI Black	-0.021 (0.014)	0.125	-0.039 (0.013)*	< 0.005
Approach * PI Black	0.045 (0.012)*	< 0.005	0.031 (0.011)	0.006
Environment * PI Black	-0.009 (0.018)	0.630	0.010 (0.018)	0.584
Random Effects				
Reviewer Intercepts Std. Dev.	0.288		0.274	
PI Intercepts Std. Dev.	0.092		0.089	
SRG Intercepts Std. Dev.	0.088		0.085	
Residual Variability Std. Dev.	0.562		0.565	

Public-use data set: Preliminary criterion, race, commensuration (race-criterion interaction) coefficients, and variance components estimates for preliminary overall impact scores on $n = 7471$ reviews of 2566 applications (matched subset) and $n = 8595$ reviews of 3045 applications (random subset). Control variables (coefficient estimates are not shown) are the matching variables. Significance * is reported for $p < .005$.

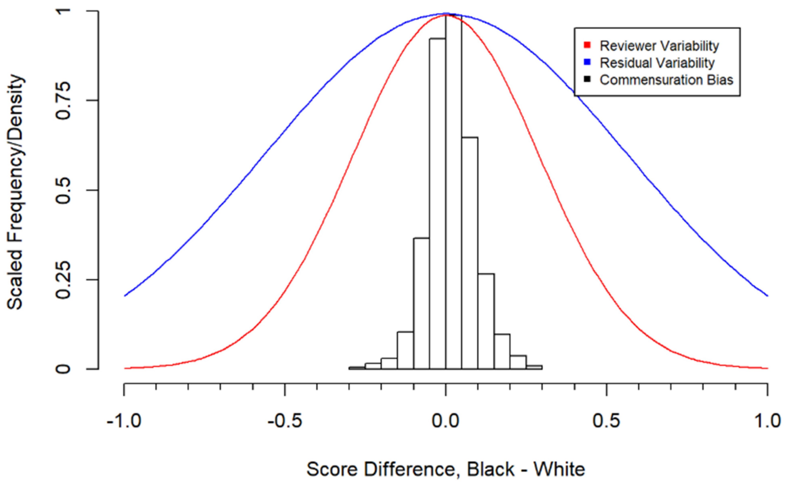

Figure S3: Public-use data set: Distribution of estimated expected preliminary overall impact score differences due to commensuration (histogram) and distributions of reviewer intercepts (red line) and model residuals (blue line), under the **matched subset commensuration model** (Table S10).

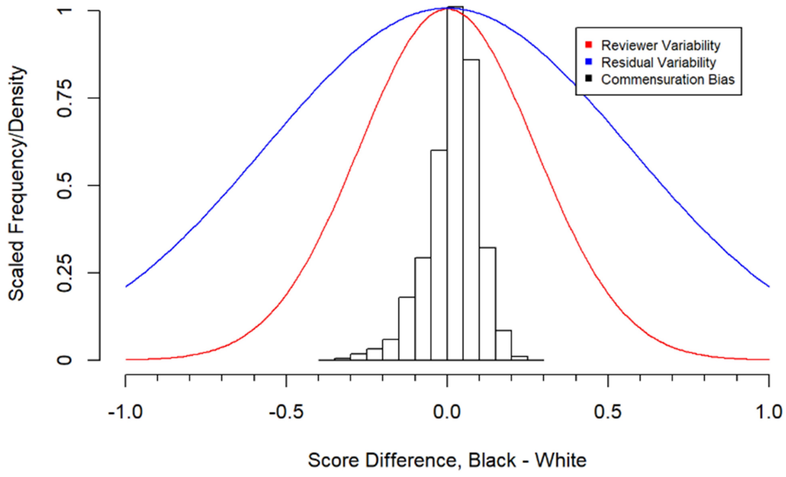

Figure S4: Public-use data set: Distribution of estimated expected preliminary overall impact score differences due to commensuration (histogram) and distributions of reviewer intercepts (red line) and model residuals (blue line), under the **random subset commensuration model** (Table S10).

REFERENCES AND NOTES

1. National Research Council, *Research Universities and the Future of America: Ten Breakthrough Actions Vital to Our Nation's Prosperity and Security* (National Research Council, 2012).

2. National Academy of Engineering, *Engineering Research and America's Future: Meeting the Challenges of a Global Economy* (National Academy of Engineering, 2005).

3. National Academy of Sciences, National Academy of Engineering, and and Institute of Medicine, *Expanding Underrepresented Minority Participation: America's Science and Technology Talent at the Crossroads* (National Academies Press, 2010).

4. L. A. Tabak, F. S. Collins, Weaving a richer tapestry in biomedical science. *Science* **333**, 940–941 (2011).

5. H. A. Valantine, F. S. Collins, National Institutes of Health addresses the science of diversity. *Proc. Natl. Acad. Sci. U.S.A.* **112**, 12240–12242 (2015).

6. D. K. Ginther, L. L. Haak, W. T. Schaffer, R. Kington, Are race, ethnicity, and medical school affiliation associated with NIH R01 type 1 award probability for physician investigators? *Acad. Med.* **87**, 1516–1524 (2012).

7. D. K. Ginther, S. Kahn, W. T. Schaffer, Gender, race/ethnicity, and National Institutes of Health R01 research awards: Is there evidence of a double bind for women of color? *Acad. Med.* **91**, 1098–1107 (2016).

8. D. K. Ginther, W. T. Schaffer, J. Schnell, B. Masimore, F. Liu, L. L. Haak, R. Kington, Race, ethnicity, and NIH research awards. *Science* **333**, 1015–1019 (2011).

9. D. K. Ginther, J. Basner, U. Jensen, J. Schnell, R. Kington, W. T. Schaffer, Publications as predictors of racial and ethnic differences in NIH research awards. *PLOS ONE* **13**, e0205929 (2018).

10. T. A. Hoppe, A. Litovitz, K. A. Willis, R. A. Meseroll, M. J. Perkins, B. Ian Hutchins, A. F. Davis, M. S. Lauer, H. A. Valantine, J. M. Anderson, G. M. Santangelo, Topic choice contributes to the lower rate of NIH awards to African-American/black scientists. *Sci. Adv.* **5**, eaaw7238 (2019).

11. G. Hodson, J. F. Dovidio, S. L. Gaertner, Processes in racial discrimination: Differential weighting of conflicting information. *Pers. Soc. Psychol. Bull.* **28**, 460–471 (2002).

12. M. I. Norton, S. R. Sommers, J. A. Vandello, J. M. Darley, Mixed motives and racial bias: The impact of legitimate and illegitimate criteria on decision making. *Psychol. Public Policy Law* **12**, 36–55 (2006).

13. M. I. Norton, J. A. Vandello, J. M. Darley, Casuistry and social category bias. *J. Pers. Soc. Psychol.* **87**, 817–831 (2004).

14. E. Uhlmann, G. L. Cohen, Constructed criteria: Redefining merit to justify discrimination. *Psychol. Sci.* **16**, 474–480 (2005).
15. E. L. Uhlmann, G. L. Cohen, “I think it, therefore it’s true”: Effects of self-perceived objectivity on hiring discrimination. *Organ. Behav. Hum. Decis. Process.* **104**, 207–223 (2007).
16. W. Thorngate, R. M. Dawes, M. Foddy, *Judging Merit* (Psychology Press, 2009).
17. D. Kahneman, *Thinking, Fast and Slow Farrar* (Straus and Giroux, ed. 1, 2013).
18. NIH Staff, *Get a Handle on Changes from the Enhancing Peer Review Process* (NIH, 2009).
19. NIH Staff, *Scoring Guidance* (NIH, 2016).
20. NIH Staff, *Scoring System and Procedure* (NIH, 2012).
21. NIH Staff, *Integrated Review Groups | NIH Center for Scientific Review* (NIH, 2019).
22. NIH Staff, *Peer Review* (NIH, 2018).
23. M. D. Lindner, A. Vancea, M. C. Chen, G. Chacko, NIH peer review: Scored review criteria and overall impact. *Am. J. Eval.* **37**, 238–249 (2016).
24. C. J. Lee. Commensuration bias in peer review. *Philos. Sci.* **82**, 1272–1283 (2015).
25. White House Staff, *Implementation of Federal Prize Authority: Fiscal Year 2014 Progress Report* (Obama White House, 2015).
26. W. N. Espeland, M. L. Stevens, Commensuration as a social process. *Annu. Rev. Sociol.* **24**, 313–343 (1998).
27. NIH Staff, *All Other CSR Special Emphasis Panels | NIH Center for Scientific Review* (NIH, 2019).
28. NIH Staff, *List of NIH Institutes, Centers, and Offices* (NIH, 2015).
29. M. K. Eblen, R. M. Wagner, D. RoyChowdhury, K. C. Patel, K. Pearson, How criterion scores predict the overall impact score and funding outcomes for National Institutes of Health peer-reviewed applications. *PLOS ONE* **11**, e0155060 (2016).
30. S. M. Iacus, G. King, G. Porro, Causal Inference without balance checking: Coarsened exact matching. *Polit. Anal.* **20**, 1–24 (2012).
31. E. Erosheva, E. C. Walton, D. T. Takeuchi, Self-rated health among foreign- and US-born asian americans: A test of comparability. *Med. Care* **45**, 80–87 (2007).
32. D. E. Ho, K. Imai, G. King, E. A. Stuart, Matching as nonparametric preprocessing for reducing model dependence in parametric causal inference. *Polit. Anal.* **15**, 199–236 (2007).
33. L. L. Hargens, J. R. Herting. Neglected considerations in the analysis of agreement among journal referees. *Scientometrics* **19**, 91–106 (1990).
34. C. J. Lee. A kuhnian critique of psychometric research on peer review. *Philos. Sci.* **79**, 859–870 (2012).

35. H. Goldstein, *Multilevel Statistical Models* (John Wiley & Sons, 2011).
36. S. W. Raudenbush, A. S. Bryk, *Hierarchical Linear Models: Applications and Data Analysis Methods* (SAGE, 2002).
37. D. J. Benjamin, J. O. Berger, M. Johannesson, B. A. Nosek, E. J. Wagenmakers, R. Berk, K. A. Bollen, B. Brembs, L. Brown, C. Camerer, D. Cesarini, C. D. Chambers, M. Clyde, T. D. Cook, P. De Boeck, Z. Dienes, A. Dreber, K. Easwaran, C. Efferson, E. Fehr, F. Fidler, A. P. Field, M. Forster, E. I. George, R. Gonzalez, S. Goodman, E. Green, D. P. Green, A. G. Greenwald, J. D. Hadfield, L. V. Hedges, L. Held, T. Hua Ho, H. Hoijtink, D. J. Hruschka, K. Imai, G. Imbens, JP. A. Ioannidis, M. Jeon, J. H. Jones, M. Kirchler, D. Laibson, J. List, R. Little, A. Lupia, E. Machery, S. E. Maxwell, M. Mc Carthy, D. A. Moore, S. L. Morgan, M. Munafó, S. Nakagawa, B. Nyhan, T. H. Parker, L. Pericchi, M. Perugini, J. Rouder, J. Rousseau, V. Savalei, F. D. Schönbrodt, T. Sellke, B. Sinclair, D. Tingley, T. Van Zandt, S. Vazire, D. J. Watts, C. Winship, R. L. Wolpert, Y. Xie, C. Young, J. Zinman, V. E. Johnson, Redefine statistical significance. *Nat. Hum. Behav.* **2**, 6–10 (2018).
38. R. L. Fleurence, L. P. Forsythe, M. Lauer, J. Rotter, J. P. Ioannidis, A. Beal, L. Frank, J. V. Selby, Engaging patients and stakeholders in research proposal review: The patient-centered outcomes research institute. *Ann. Intern. Med.* **161**, 122–130 (2014).
39. D. Kahneman, G. Klein. Conditions for intuitive expertise: A failure to disagree. *Am. Psychol.* **64**, 515–526 (2009).
40. A. D. Higginson, M. R. Munafò, Current incentives for scientists lead to underpowered studies with erroneous conclusions. *PLOS Biol.* **14**, e2000995 (2016).
41. M. D. Lindner, K. D. Torralba, N. A. Khan, Scientific productivity: An exploratory study of metrics and incentives. *PLOS ONE* **13**, e0195321 (2018).
42. P. E. Smaldino, R. McElreath, The natural selection of bad science. *R. Soc. Open Sci.* **3**, 160384 (2016).
43. J. Wang, R. Veugelers, P. Stephan. Bias against novelty in science: A cautionary tale for users of bibliometric indicators. *Res. Policy* **46**, 1416–1436 (2017).
44. E. T. Warner, R. Carapinha, G. M. Weber, E. V. Hill, J. Y. Reede, Faculty promotion and attrition: The importance of coauthor network reach at an academic medical center. *J. Gen. Intern. Med.* **31**, 60–67 (2016).
45. A. G. Greenwald, D. E. McGhee, J. L. Schwartz, Measuring individual differences in implicit cognition: The implicit association test. *J. Pers. Soc. Psychol.* **74**, 1464–1480 (1998).

46. J. F. Dovidio, S. L. Gaertner, Aversive racism and selection decisions: 1989 and 1999. *Psychol. Sci.* **11**, 315–319 (2000).
47. F. D. Blau, J. M. Currie, R. T. A. Croson, D. K. Ginther, Can mentoring help female assistant professors? Interim results from a randomized trial. *Am. Econ. Rev.* **100**, 348–352 (2010).
48. NIH Staff, *Definitions of Criteria and Considerations for Research Project Grant (RPG/R01/R03/R15/R21/R34) Critiques* (NIH, 2016).
49. L. V. Hedges, Effect sizes in cluster-randomized designs. *J. Educ. Behav. Stat.* **32**, 341–370 (2007).
50. R. J. A. Little, D. B. Rubin, *Statistical Analysis with Missing Data* (John Wiley & Sons, 2019).
51. J. A. Hausman, Specification tests in econometrics. *Econometrica* **46**, 1251–1271 (1978).
